# Rapid ^13^C NMR hyperpolarization delivered from *para*-hydrogen enables the low concentration detection and quantification of sugars†
†Electronic supplementary information (ESI) available: NMR data used in this manuscript. See DOI: 10.1039/c9sc03450a


**DOI:** 10.1039/c9sc03450a

**Published:** 2019-09-24

**Authors:** Peter M. Richardson, Wissam Iali, Soumya S. Roy, Peter J. Rayner, Meghan E. Halse, Simon B. Duckett

**Affiliations:** a The Centre for Hyperpolarisation in Magnetic Resonance , Department of Chemistry , University of York , UK . Email: simon.duckett@york.ac.uk

## Abstract

Monosaccharides, such as glucose and fructose, are important to life. In this work we highlight how the rapid delivery of improved ^13^C detectability for sugars by nuclear magnetic resonance (NMR) can be achieved using the *para*-hydrogen based NMR hyperpolarization method SABRE-Relay (Signal Amplification by Reversible Exchange-Relay). The significant ^13^C signal enhancements of 250 at a high field of 9.4 T, and 3100 at a low field of 1 T, enable the detection of trace amounts of these materials as well as the quantification of their tautomeric makeup. Using studies on ^13^C and ^2^H isotopically labelled agents we demonstrate how hyperpolarization lifetime (*T*_1_) values can be extended, and how singlet states with long lifetimes can be created. The precise quantification of d-glucose-^13^C_6_-*d*_7_ at the millimolar concentration level is shown to be possible within minutes in conjunction with a linear hyperpolarized response as a function of concentration. In addition to the measurements using labelled materials, low concentration detection is also illustrated for millimolar samples with natural abundance ^13^C where isomeric form quantification can be achieved with a single transient.

## Abstract 2

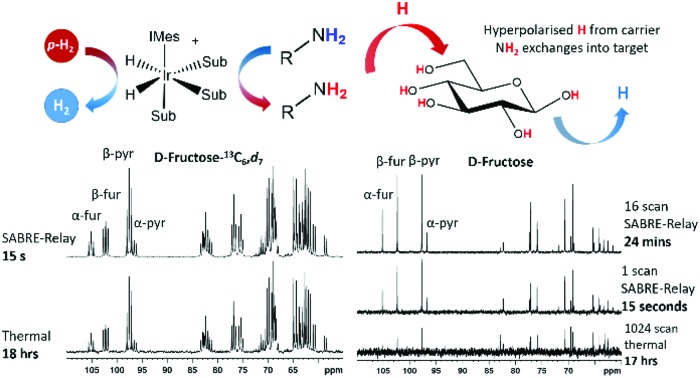
The monosaccharides glucose and fructose are rapidly detected and quantified by ^13^C NMR in conjunction with the hyperpolarisation method signal amplification by reversible exchange-relay.

## Introduction

Saccharides play an integral role in metabolism in the body with glycolysis being one of the most well studied metabolic pathways.^1^ Anomalies in this pathway are characteristic signs of several pathological conditions including cancer,^2^,^3^ Alzheimer's disease,^4^ coronary artery disease,^5^ and septic shock.^6^ Consequently, the quantification of the metabolites involved here reflects a potential route to diagnose these conditions.

In addition to probing these diagnostic biomarkers, there is significant research in determining the concentration and the tautomeric distributions of saccharides in solution.^7^,^8^ Both glucose and fructose can in fact exist as multiple tautomers, as either α or β isomers with keto (open chain), pyranose (six-membered ring) or furanose (five-membered ring) forms.^9^–^11^ The relative tautomeric composition of fructose is of interest as it relates to the overall sweetness of the sugar, where d-fructopyranose is significantly sweeter than the d-fructofuranose form.^12^,^13^ The sweetness of fructose can be manipulated by variation of concentration, pH, temperature and solvent to reduce the furanose component and thus increase sweetness.^7^,^14^

Nuclear magnetic resonance (NMR) is a very powerful analytical tool that has been used extensively to monitor these changes.^15^ However, there are certain limitations associated with this technique. Firstly, NMR is inherently insensitive, as it relies on the population difference between nuclear spin state orientations as defined by the Boltzmann distribution. Consequently, at 9.4 T (400 MHz) only 1 in 31 000 ^1^H nuclei contribute positively to the detected response. Typically, this is alleviated by employing strong superconducting magnets, which provide improved signal strengths and increased spectral dispersion. Secondly, ^1^H NMR is limited by the dynamic range of the digitizer and while all the different saccharide isomers respond differently, highly complex, often overlapping spectra result with both weak and strong components.^8^,^16^,^17^ To optimally track this distribution, and any associated chemical change, it would be sensible to use ^13^C-NMR because of the broader chemical shift range of the sugar response.^18^ However, ^13^C NMR has a low gyromagnetic ratio and low natural abundance (1.1%) which normally means isotopically labelled compounds or high concentrations and long acquisition times are needed, which can lead to expensive analysis.

One route to overcoming the issue of sensitivity in NMR is hyperpolarization, which is defined as any significant deviation away from the Boltzmann distribution of spin populations which results in strongly enhanced NMR signals.^19^–^21^ There are several different hyperpolarization methods including dynamic nuclear polarization (DNP),^22^ dissolution-DNP (D-DNP),^23^,^24^ spin exchange optical pumping,^25^–^27^ brute force,^28^ and *para*-hydrogen induced hyperpolarization (PHIP).^29^–^31^ These techniques have been extensively used for the polarization of biomolecules.^32^–^34^ D-DNP has become a particularly popular hyperpolarization technique, which has proven to be applicable for use in metabolic magnetic resonance imaging (MRI).^24^,^35^–^38^ D-DNP has also been shown to provide significant signal gains that allow the monitoring of glycolysis in cancerous cells.^39^,^40^ However, there are several drawbacks to this technique. Firstly, it often uses significant amounts of isotopically labelled samples which can be costly. Secondly, the time taken to polarize the molecule is typically on the order of hours. Thirdly it is a single-shot measurement, meaning that if the experiment fails both the result and the materials are lost. Here we demonstrate the viability of using the *para*-hydrogen (*p*-H_2_) based hyperpolarization technique, signal amplification by reversible exchange (SABRE) to circumvent some of these issues.

*Para*-hydrogen is a spin isomer of H_2_, which is normally present at 25%, with the remainder being *ortho*-hydrogen.^41^ The conversion of *ortho*-H_2_ to *p*-H_2_ is relatively easy and cost effective. It requires the gas to be cooled and passed over a paramagnetic catalyst. With the paramagnetic catalyst removed, the *p*-H_2_ enriched gas can subsequently be warmed and stored for long periods without loss of enrichment. In order to unlock the potential of *p*-H_2_, its symmetry must first be broken through a chemical reaction. Originally, this was achieved by the hydrogenation of a substrate of interest which placed nuclei that were in *p*-H_2_ into the product.^42^–^45^ In fact, this process has been exemplified for use with certain tagged glucose derivatives.^46^ However, the downside to this method is that it is not reversible due to the permanent nature of the chemical change. Conversely, SABRE is a reversible catalytic process that involves the transfer of nuclear spin order from the former *p*-H_2_ molecule into a target substrate.^31^,^47^ This is achieved through its reversible ligation to a metal center, with the resulting active complex's asymmetry allowing transfer of polarization from *p*-H_2_ derived hydride nuclei to the bound substrate. Substrate dissociation then leads to a build-up hyperpolarized substrate in free solution without change in its chemical identity. The SABRE technique is therefore completely reversible with the addition of fresh *p*-H_2_. The polarization it delivers is typically established on the order of tens of seconds, and is achieved outside the spectrometer in a small polarization transfer field (PTF) of around 0–10 mT to satisfy the necessary resonance conditions for polarization transfer.^41^,^48^ Since the polarization is produced *ex situ* it is also independent of detection field, which means detection can occur on significantly cheaper benchtop devices.^49^–^51^

**Fig. 1:**
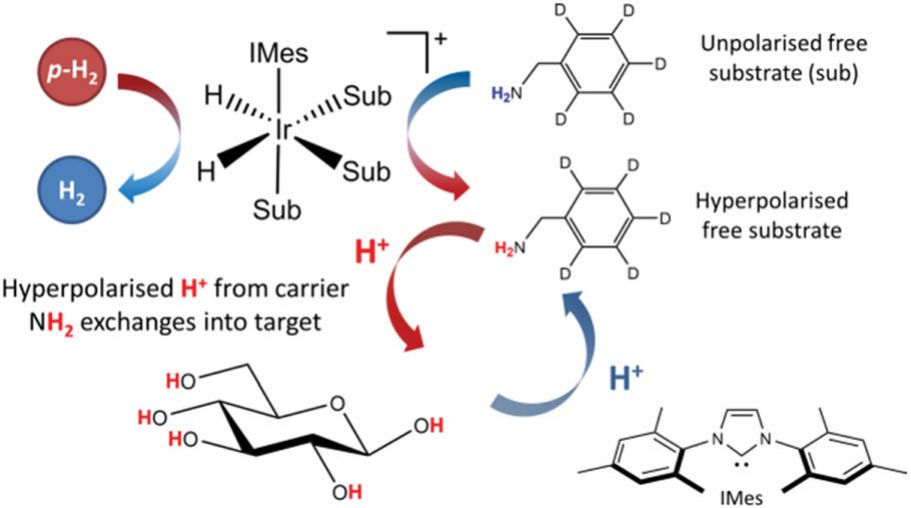
Schematic for the SABRE-Relay process, with a carrier molecule first undergoing SABRE and subsequently passing polarization into glucose *via* proton exchange.

The applicability of SABRE is not limited to ^1^H NMR and can be applied to many different nuclei such as ^13^C,^52^–^54^ ^19^F,^55^,^56^ and ^15^N,^57^–^59^ among others.^60^,^61^ In the case of ^13^C and ^15^N, the resonance condition for SABRE occurs at much lower magnetic fields, so PTFs of around 5 mG are required for the direct transfer of polarization from the catalysts hydride ligands.^57^ A requirement for SABRE is that reversible ligation of the substrate to the metal center of the catalyst takes place on a suitable timescale, a parameter that can be tuned by varying a stabilizing N-heterocyclic carbene (NHC) on the catalyst.^62^ Although the SABRE technique has been demonstrated to work very effectively with N-heterocyclic substrates, such as pyridine derivatives, this provides a limited selection of target molecules. An extension to the SABRE method termed SABRE-Relay provides a route to hyperpolarize a broader range of materials.^63^ In the SABRE-Relay methodology a carrier substrate is chosen that contains a functional group with an exchangeable proton, which once hyperpolarized in free solution can undergo proton exchange with a secondary target such that it can become hyperpolarized, a schematic of this process is depicted in Fig. 1. Currently this mechanism has been shown to extend the range of molecules amenable to SABRE to include alcohols, amines, amides, carboxylic acids, phosphates, and carbonates.^64^–^67^

In this work, we illustrate the first in depth study of the hyperpolarization of ^13^C NMR glucose and fructose *via* the SABRE-Relay method. We discuss the enhancement levels that can be obtained and demonstrate the viability of this simple method for tautomeric analysis and quantification of low mM concentrations of glucose.

## Experimental

### Sample preparation

Samples were prepared using 4.8 mM of the SABRE pre-catalyst [IrCl(COD)(NHC)] (COD = 1,5-cyclooctadiene), where all catalysts were synthesized in house. Various catalysts with different NHC's were tested, with 23.8 mM of benzyl-*d*_7_-amine (*d*_7_-BnNH_2_, purchased from CDN Isotopes) in dichloromethane-*d*_2_ (DCM-*d*_2_). This solution was subsequently added to a NMR tube fitted with a Young's valve (GPE Scientific) and degassed using a freeze–pump–thaw procedure in liquid nitrogen.^68^ H_2_ gas at 4 bar (absolute) was added to the tube and shaken for 10 seconds to promote the gas to dissolve into solution. The catalyst activation for these samples occurred over a longer timescale than the typical pyridine-based SABRE targets, therefore the samples were left at room temperature overnight to allow complete activation to occur. The catalyst is fully activated once the COD ligand has converted to cyclooctane, leaving a catalyst of general form [Ir(NHC)H_2_(BnNH_2_)_3_]Cl.^69^ After activation, 2.5 mg of fructose/glucose was dissolved in 0.25 mL of dimethylformamide (DMF) and subsequently added to the solution containing the catalyst inside an air-free glovebox.

In this study a variety of isotopically labelled versions of both glucose and fructose were analysed using the SABRE-Relay method. Typically 2.5 mg of glucose or fructose was used in an overall sample volume of 0.65 mL. We also note from previous observations using SABRE-Relay that the amount of residual water in the sample needs to be kept to a minimum due to leaching of polarization from the target analytes. As DMF is hydroscopic, anhydrous DMF was sourced from Acros Organics and subsequently stored in a glovebox. Upon removal of the nitrogen gas, by replacing it with *p*-H_2_ significant enhancements of the glucose/fructose signals under SABRE-Relay resulted. The *p*-H_2_ was produced with a bespoke generator, operating at 28 K using an iron oxide based paramagnetic catalyst to allow efficient conversion to the *para*-state. This generator has been detailed elsewhere.^65^

### NMR spectrometers and experiments

NMR spectra were measured either on a 400 MHz Bruker Avance III spectrometer using a BBI probe or a 43 MHz (1 T) Magritek Spinsolve Carbon benchtop NMR spectrometer. The hyperpolarized spectra shown were all acquired with a simple 90° pulse and acquire protocol either with, or without, ^1^H/^2^H decoupling as described in the text. The polarization transfer field (PTF) used was 60.6 G (unless otherwise stated), and achieved using a hand-held magnet array.^70^ The hyperpolarization lifetime measurements were determined in two ways. Firstly, a multiple step manual hyperpolarization method, where the sample was filled with fresh *p*-H_2_ and shaken for 10 seconds at 60.6 G before being transferred to the spectrometer for detection. A variable delay time was written into the pulse sequence and applied following insertion and prior to each measurement. This delay time was defined as the time after insertion. This process was repeated for several delay times to encode relaxation. Secondly, a single-shot procedure was used which relies on an increasing variable flip angle sequence to encode relaxation.^71^ This provides a fast method to measure magnetization lifetime, however, since the initial pulse is 15° the maximum signal that can be achieved is around 25% when compared to the multistep method. The single shot approach was not used for the lower field measurements due to insufficient signal to noise. The total experiment time was around 30 minutes for the manual shaking method and around 2 minutes for the single shot measurement.

## Results and discussion

### Hyperpolarization of glucose

**Fig. 2:**
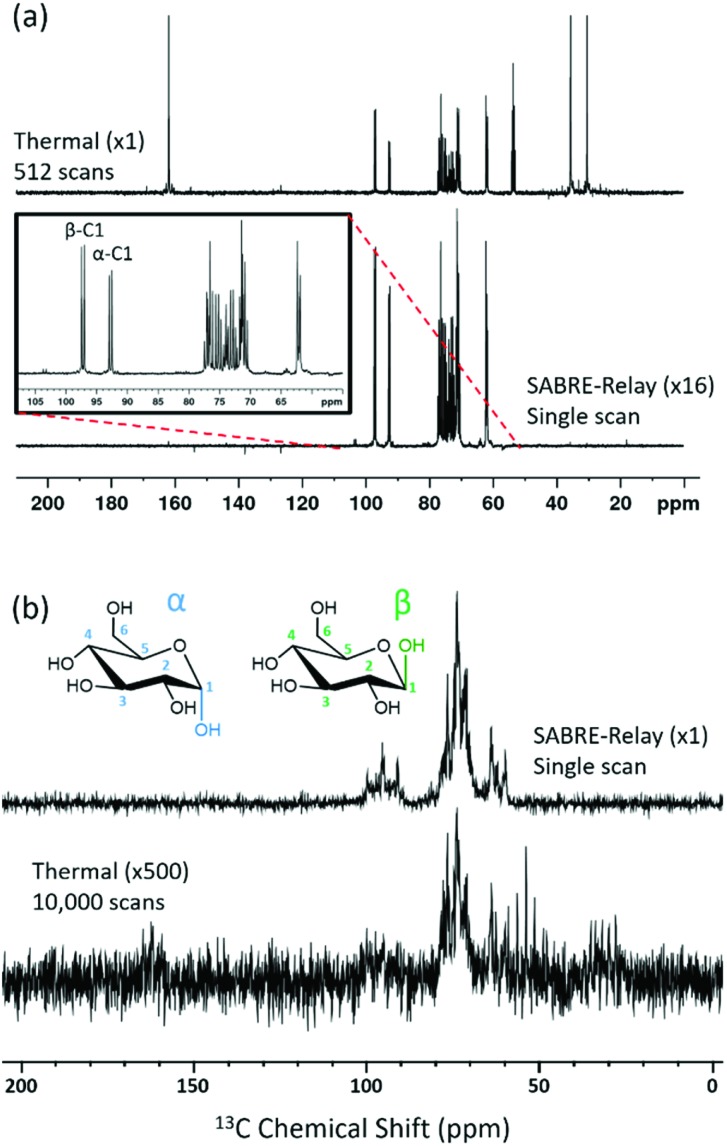
(a) Single scan ^13^C{^1^H} hyperpolarized NMR spectrum (bottom spectrum, ×16) and a thermal reference NMR spectrum (top spectrum) of d-glucose ^13^C_6_ acquired with 512 transients, measured at 9.4 T (400 MHz). (b) Analogous 1 T (43 MHz) measurement; the thermal reference spectrum now needed 10 000 transients (bottom spectrum, which has also been vertically scaled ×500 relative to the upper trace). The structures of the two isomeric forms of glucose (α and β) that are detected are shown inset.

The SABRE-Relay technique has been shown to hyperpolarize a range of analytes and works particularly well for alcohols.^64^ In principle, this technique can be extended to any motif that includes a proton which exchanges with the carrier molecule, typically an amine, on a suitable timescale. When d-glucose-^13^C_6_ was examined with SABRE-Relay, using [IrCl(COD)(IMes)] as the precatalyst and *d*_7_-BnNH_2_ as the carrier agent, a ^13^C{^1^H} hyperpolarized NMR spectrum could be acquired in a single scan as shown in Fig. 2a (bottom spectrum) for a 400 MHz spectrometer. This spectrum is crowded due to the presence of one-bond ^13^C–^13^C couplings of approximately 45 Hz.

A thermally polarized ^13^C NMR spectrum, acquired in 512 scans (128 min), is shown in Fig. 2a for comparison (top spectrum). This allowed an estimate for the enhancement factor of 99 ± 5 (0.3% polarization) for the ^13^C signals to be determined. Fig. 2b shows an analogous pair of spectra acquired on a 1 T benchtop NMR system. Due to the reduced detection field, the thermal reference spectrum required 10 000 scans (2500 min). It can be readily observed from Fig. 2b that there is a reduction in chemical shift dispersion on moving to lower magnetic field which exacerbates spectral crowding. However, the level of NMR signal enhancement observed on the benchtop NMR is now estimated as 3100 ± 160 which is *ca.* 1.1% polarization. Based on the reduction in the magnetic field used for detection, the low-field enhancement factor is expected to be only 940 (9.4 times larger than at high field). Therefore the 1 T benchtop result is about 3.3 times higher than expected. This could be attributed to a shorter sample transfer time; typically, a high-field transfer takes around 4.0 ± 0.5 s, whereas because the benchtop spectrometer is more easily accessed it takes just 3.0 ± 0.5 s for transfer. If the *T*_1_ is sufficiently short then this small difference could become significant. Additionally, the sample that is transferred into the 9.4 T spectrometer will experience a larger field gradient than that which is transferred into the 1 T spectrometer due to the differing stray fields. It is possible that this can lead to increased polarization loss during the transfer step and result in the reduced signal gains that are observed at 9.4 T.

### Hyperpolarization lifetime

**Fig. 3:**
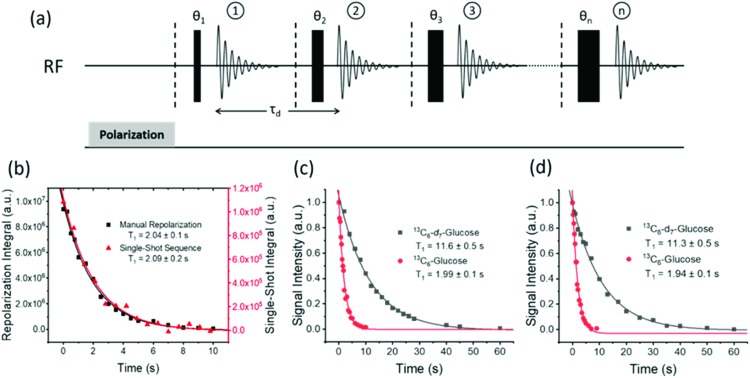
(a) Single-shot pulse sequence used to measure the hyperpolarized lifetime (*T*_1_) values, in this study; 15 points were measured per experiment with a starting angle of 15° which gives 25.8% of the polarization for each point. (b) Comparison of the repolarization and single-shot methods of measuring the hyperpolarized *T*_1_ which give the same decay, although the single shot measurement involves significantly less signal intensity. A comparison of hyperpolarization lifetimes between d-glucose-^13^C_6_-*d*_7_ and d-glucose-^13^C_6_ is displayed at (c) 9.4 T and (d) 1 T, using the manual repolarization method in each case. All samples contained 2.5 mg of agent, 4.8 mM of catalyst **3** with 23.8 mM benzyl-*d*_7_-amine in 0.65 mL DCM-*d*_2_ : DMF (1.6 : 1).

The hyperpolarized signal lifetime reflects the standard longitudinal NMR relaxation time, *T*_1_, in these systems and should be independent of the starting polarization level (as exemplified in the ESI†). In this study we have employed two methods to determine this. Firstly, due to the reversible nature of SABRE-Relay, it was possible to repolarise a sample to complete an array of measurements with a variable delay before data acquisition to allow sample relaxation to take place following transfer into the magnet. In this way, relaxation is encoded in the detection field. Secondly, a single-shot sequence was used which employs increasing pulse durations to take an equal proportion of magnetization with each acquisition as shown schematically in Fig. 3a.^71^ A benefit of this approach is that it can be completed following a single hyperpolarization period. However, a drawback is that the measurement's signal to noise is reduced relative to the multistep procedure. At high-field the SABRE-Relay hyperpolarization method provides sufficient signal to allow the single shot sequence to be used. Conversely, when acquiring data on the lower field 1 T spectrometer, the signal to noise is not sufficient for the single-shot method and therefore the repolarization technique is used.

Fig. 3b shows a decay curve for a sample of d-glucose-^13^C_6_ which has been measured using both the repolarization method (black squares) and the single-shot sequence (red triangles). The values obtained for this sample were 2.04 ± 0.1 s and 2.09 ± 0.2 s for the repolarization and single-shot sequences respectively. We note that the observed hyperpolarized *T*_1_ values measured here are relatively short, which may limit their use in some practical applications. However, the hyperpolarized *T*_1_ value reported in the literature when using the D-DNP hyperpolarization method is around 12 s for a sample of d-glucose-^13^C_6_-*d*_7_.^35^Fig. 3c shows a comparison of the SABRE-Relay hyperpolarization lifetimes measured at 9.4 T for the deuterated (black squares) and non-deuterated (red circles) analogues of d-glucose-^13^C_6_. It can be readily seen that the lifetimes are significantly increased from 1.99 ± 0.1 s to 11.6 ± 0.5 s in the presence of deuterium. Analogous measurements performed at 1 T are shown in Fig. 3d which reveal that the observed hyperpolarization lifetime of both forms of d-glucose are comparable in the two detection fields. The hyperpolarization lifetime obtained here of 11.6 ± 0.5 s is in agreement with that measured using the D-DNP method.^35^ This result is noteworthy as in the standard SABRE approach, the *T*_1_ of the target molecule is greatly reduced in the presence of the active SABRE catalyst.^72^ This is due to the large difference in the *T*_1_ of the free molecule in solution and when it is bound to the catalyst. As these two forms are in rapid chemical exchange, the observed lifetime is a weighted average of the *T*_1_ values in the two forms.^72^ In the case of SABRE-Relay, the target substrate does not bind to the catalyst, and therefore the catalyst is not expected to have any significant effect on the relaxation of the glucose in free solution. In fact, as measured using standard NMR, the *T*_1_ values of the benzylamine and glucose combination in free solution increase slightly in the presence of the active SABRE catalyst (more details can be found in the ESI†). We hypothesize that this increase in *T*_1_ is due to a reduction in concentration of NH protons available in solution for exchange with glucose due to the binding of benzylamine to the catalyst.

### Selective ^13^C and ^2^H labelling

**Fig. 4:**
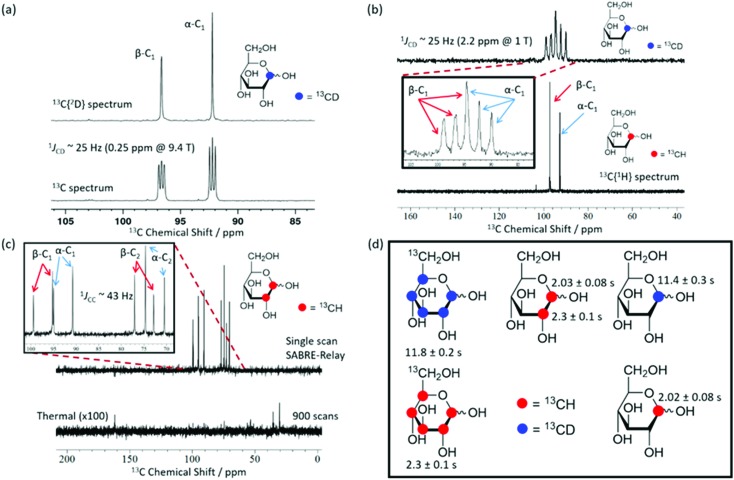
SABRE-Relay hyperpolarized ^13^C NMR spectra. (a) ^13^C{^2^H} NMR spectrum (top) and a ^13^C NMR spectrum (bottom) of 2.5 mg (21.1 mM) of mM d-glucose-1-^13^C_1_-*d*_1_ (structure inset) with 4.8 mM of **3** and 23.8 mM benzyl-*d*_7_-amine in 0.65 mL DCM-*d*_2_ : DMF (1.6 : 1) measured at 9.4 T. (b) Corresponding results showing a ^13^C spectrum of d-glucose-1-^13^C_1_-*d*_1_ (top) and a ^13^C{^1^H} spectrum of d-glucose-1-^13^C_1_ (bottom) measured at 1 T. The inset spectrum reflects the boxed region of interest and highlights the presence of two isomers whose signals overlap. (c) Hyperpolarized (top) and 900 scan thermal reference scan (bottom, ×100 scaling) for a 21.1 mM d-glucose-1,2-^13^C_2_ sample with 4.8 mM of **3** and 23.8 mM benzyl-*d*_7_-amine in DCM-*d*_2_ : DMF (1.6 : 1) (0.6 mL) acquired at 1 T. The inset of (c) shows the region of interest and demonstrates that both isomers can be distinguished at 1 T. (d) Structures of the glucose variants used in this study along with their corresponding hyperpolarized *T*_1_ values at 1 T. In the case of the ^13^C_6_ variants all peaks were analyzed together due to spectral overlap.

Using SABRE-Relay, it is possible to hyperpolarize d-glucose-^13^C_6_ and d-glucose-^13^C_6_-*d*_7_ and observe their ^13^C NMR signals in just a few tens of seconds even at 1 T. However, the resultant ^13^C NMR spectra contain multiple overlapping resonances due to the large ^13^C–^13^C couplings these systems exhibit. Simplification of these spectra could be achieved with homonuclear decoupling techniques such as pure shift.^73^ However, the gain in resolution is typically associated with a significant reduction in sensitivity. An alternative strategy is to simplify the target molecule to negate this effect. Fig. 4a shows a hyperpolarized ^13^C NMR spectrum of d-glucose-1-^13^C_1_-*d*_1_ at a 21.1 mM loading (sample used 4.8 mM [IrCl(COD)(IMes)] and 23.8 mM *d*_7_-BnNH_2_ in DCM-*d*_2_ : DMF (1.6 : 1)) acquired at 9.4 T. This spectrum is significantly simpler than those of Fig. 2b. Analogous spectra are shown in Fig. 4b at 1 T. For the spectra with a retained ^2^H coupling, triplets appear where ^1^*J*_CD_ is ∼25 Hz. At 1 T, this coupling corresponds to around 2.2 ppm, which means there is now overlap between the multiplets of the two isomers whereas at 9.4 T it is 0.25 ppm and peak separation is achieved. However, the resulting NMR spectra confirm isomer differentiation is possible even at 1 T, which when coupled with the longer *T*_1_ lifetimes means a range of applications might be envisaged for this technique.

Fig. 4c shows a hyperpolarized ^13^C{^1^H} NMR spectrum (top) and the corresponding long acquisition thermal reference NMR spectrum (bottom) for an analogous sample of d-glucose-1,2-^13^C_2_ that were acquired at 1 T. This sample also retains full spectral clarity despite the introduction of a second ^13^C resonance. The inset of Fig. 4c shows the region of interest in more detail. There are four doublets in the spectrum, one for each of the two labelled ^13^C resonances and their two isomeric forms (structures can be seen in Fig. 2b). This ^13^C labelling variation can be used to create an NMR spin singlet and will be considered in the next section. Fig. 4d shows all of the isotopologues of glucose used in this study where a red circle represents a ^13^CH site and a blue circle represents a ^13^CD site. The relevant values of the *T*_1_ relaxation times for these ^13^C sites are displayed alongside the corresponding structure. In the case of the fully labelled d-glucose-^13^C_6_ and d-glucose-^13^C_6_-*d*_7_ due to peak overlap an ensemble average is presented. It can be concluded from these values that when a ^13^C nucleus is situated next to ^1^H the resulting lifetime is around 2 seconds, whereas when it is next to a ^2^H nucleus the lifetime is around 11 seconds at 9.4 T. There is little difference between the lifetimes of fully labelled d-glucose-^13^C_6_-*d*_7_ and d-glucose-1-^13^C,1-*d* which are 11.8 ± 0.4 s and 11.4 ± 0.3 s respectively, highlighting that there seems to be negligible relaxation coming from the ^1^H nuclei that are not directly bound to the ^13^C under investigation.

We conclude that selective labelling can be useful in reducing spectral clutter and improving the resulting peak amplification under SABRE-Relay. Consequently, the observed signal to noise ratios increase which means that any analytical assessment will be more reliable.

### Singlet formation

**Fig. 5:**
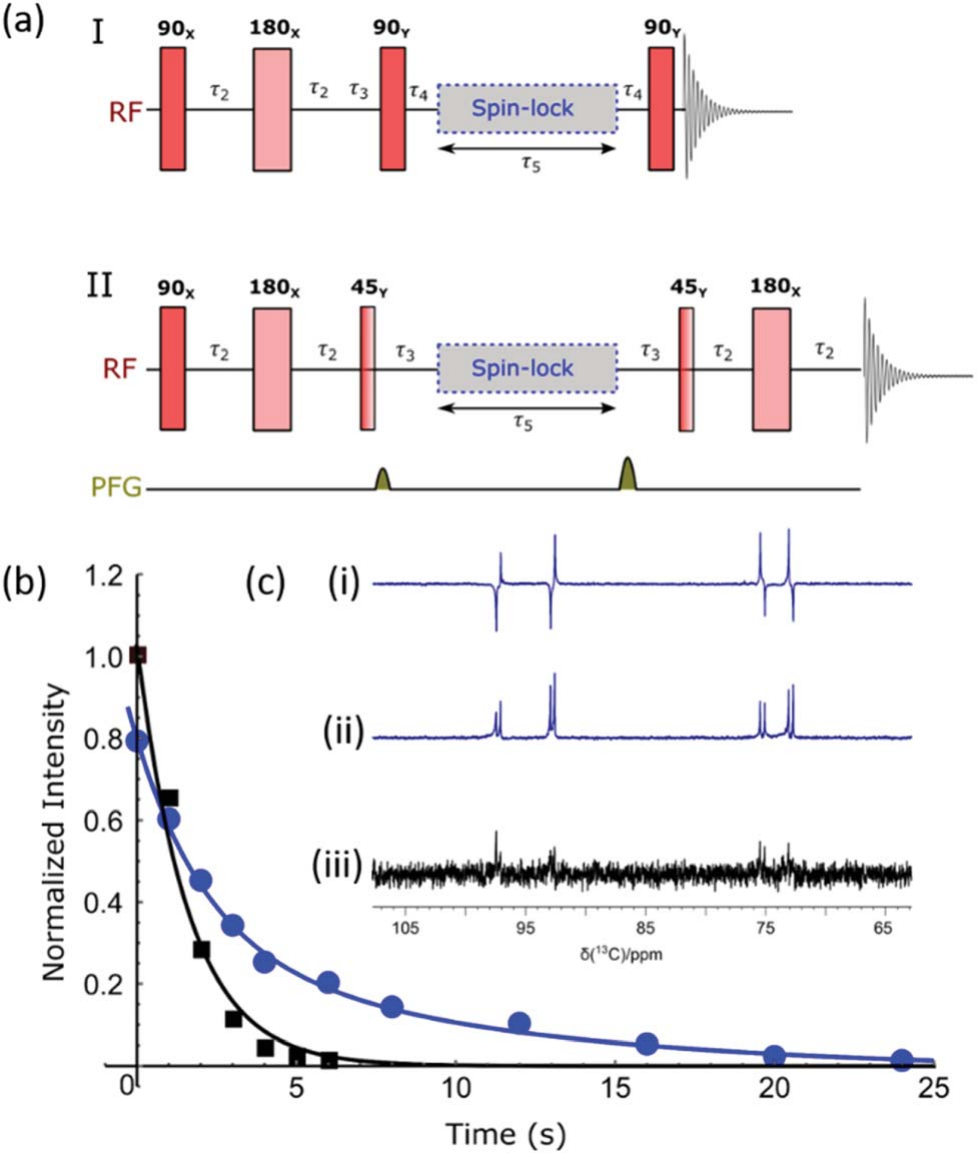
(a) Pulse sequences used to create and then probe singlet spin order. I produces an anti-phase magnetization, while II refocuses this response to create the more usual in-phase signal; delays are as defined in text. (b) Normalized signal intensities plotted against sample storage time to determine the lifetime of singlet state (9.9 ± 2.3 s) using method II. The sample was repolarized between each data point (blue curve); longitudinal magnetization decays with a lifetime of 1.6 ± 0.2 s. (c) Region of these ^13^C{^1^H} NMR spectra showing the hyperpolarized signal derived from d-glucose-1,2-^13^C_2_ – (i) method I, (ii) method II and (iii) signal acquired from longitudinal thermal magnetization.

As previously mentioned, the doubly labelled material offers a route to access a nuclear spin singlet. This type of spin state can be created through the use of RF (radio frequency) excitation and such states may have long lifetimes.^74^,^75^ Fig. 5a shows two different pulse sequences which can be used to achieve singlet order in the sample given that there are two coupled spins with a chemical shift difference which is typically less than 500 Hz. Method I converts this spin order into anti-phase magnetization for detection,^76^ and method II has been modified to include a refocusing step to convert this into an in-phase signal.^77^ The NMR spectra resulting from the application of these methods, as well as the corresponding thermal derived result, are shown in Fig. 5c. Antiphase behaviour is seen with signals arising from both the α and β isomers of glucose. The addition of pulse field gradients (PFG) to method II resulted in a more robust measurement.^77^ The delays used in the pulse sequence are defined as per the spin network, where *τ*_2_ = 1/4*J*_IS_, *τ*_3_ = 1/2Δ*ν*_IS_, *τ*_4_ = 1/4Δ*ν*_IS_, with *J*_IS_ and Δ*ν*_IS_ representing the scalar coupling constant and chemical shift difference between spin I and spin S respectively. Fig. 5b shows how the response of method II varies with the *τ*_5_ delay (blue circles) for a sample that was repolarized before each acquisition. Analogous hyperpolarized measurements were also completed at identical times after sample introduction using a simple pulse and collect protocol (black squares) for comparison. The lifetimes resulting from fitting the exponential decays were 9.9 ± 2.3 s and 1.6 ± 0.2 s for the long-lived state and longitudinal magnetization respectively. Hence the creation of a singlet state significantly extends the lifetime of the hyperpolarized signal; we anticipate when this method is coupled with deuteration further improvements in lifetime will result.

### Optimization of SABRE-Relay

Optimization of the hyperpolarization step is essential if low concentrations of these sugars are to be detected. One immediate problem faced when optimizing this process for glucose was the presence of water in the solution which acts to waste *p*-H_2_ derived polarization through spin dilution. This was exacerbated by the need to use hydroscopic DMF as a co-solvent. Sample preparations were however completed inside a dry nitrogen filled glovebox to minimize this effect alongside dry solvents.

**Fig. 6:**
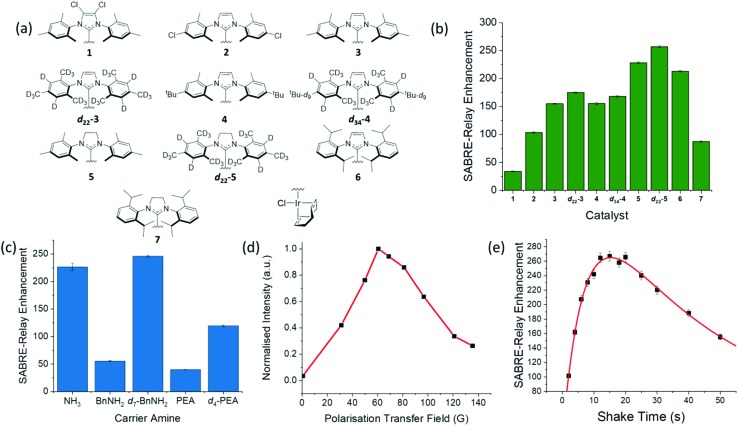
(a) Structures of the ten different catalysts used in collecting the SABRE-Relay ^13^C signal enhancements for d-glucose-^13^C_6_-*d*_7_ shown in (b); the largest enhancement was observed for ***d*_22_-5**. (c) Corresponding ^13^C signal enhancement factors achieved using pre-catalyst ***d*_22_-5** and the indicated NH containing materials ammonia (NH_3_), benzylamine (BnNH_2_), benzyl-*d*_7_-amine (*d*_7_-BnNH_2_), phenethylamine (PEA) and phenethyl-1,1,2,2,*d*_4_-amine (*d*_4_-PEA); all samples contained 10.3 mM of the NH source and 4.8 mM of ***d*_22_-5** in a DCM-*d*_2_ : DMF (1.6 : 1) solution that was shaken at 60.6 G for ten seconds. (d) Polarization transfer field dependence on the signal gain for a d-glucose-^13^C_6_-*d*_7_ sample with 4.8 mM of **3** in a DCM-*d*_2_ : DMF (1.6 : 1) solution measured at 1 T; PTF obtained using a set of bespoke Halbach arrays.^69^ (e) Signal gain as a function of shake time for a d-glucose-^13^C_6_-*d*_7_ sample using 4.8 mM of ***d*_22_-5** in a DCM-*d*_2_ : DMF (1.6 : 1) solution; a bi-exponential equation was used to fit the data which highlights the process of polarization build up *versus p*-H_2_ consumption in the NMR tube.

First the catalyst for SABRE-Relay was optimized. This involved using ten different pre-catalysts of the general form [IrCl(COD)(NHC)] (structures in Fig. 6a) with d-glucose-^13^C_6_-*d*_7_. The associated NHC's were varied to have a range of different electronic and steric effects based on previous reports.^62^Fig. 6b shows the resulting ^13^C signal enhancement factors as a function of catalyst identity, where the values were obtained relative to an external reference (see ESI†). All samples were made from a stock solution to ensure consistency between samples. The d-glucose-^13^C_6_-*d*_7_ was present at a concentration of 19.9 mM with 4.8 mM of the relevant catalyst and 23.8 mM benzyl-*d*_7_-amine. Of the non-isotopically labelled catalysts tested, **5** gave the largest signal enhancements of 225 ± 1 whilst catalyst **6** was the next highest performing. The isotopologue ***d*_22_-5** further improved the signal enhancement to 257 ± 2 which is consistent with effects of deuteration reported elsewhere.^67^,^72^ The corresponding ^2^H labelled isotopomer of **6** was unable to be synthesised however we would expect a similar improvement in glucose polarization.

In a second step, five different NH carriers were tested with ***d*_22_-5**, ammonia (NH_3_), benzylamine (BnNH_2_), benzyl-*d*_7_-amine (*d*_7_-BnNH_2_), phenethylamine (PEA) and phenethyl-1,1,2,2-*d*_4_-amine (*d*_4_-PEA). The average ^13^C signal enhancements for each amine are shown in Fig. 6c, with *d*_7_-BnNH_2_ yielding the best results.

The polarization transfer field (PTF) has proven to be crucial for SABRE.^48^,^78^ Theoretically, for direct polarization transfer to ^13^C in classic SABRE, magnetic fields of around 2.5 mG are^70^ optimal, which requires the use of mu-metal shields to negate the Earth's magnetic field.^57^,^58^ However, SABRE-Relay hyperpolarizes the glucose ^13^C nuclei *via* a polarized OH proton which is introduced into the sugar through NH/OH exchange with the carrier amine. Fig. 6d shows the SABRE-Relay enhancement on the glucose as a function of PTF, where the field is generated by permanent magnetic arrays ranging from 30–140 G,^70^ and the 0.5 G measurement used the Earth's field. It can be seen that the maximum signal enhancement factor was achieved when using a 60.6 G PTF. The transfer of hyperpolarization from glucose's hydroxyl protons to its ^13^C nuclei is predicted to take place through a mixture of low field thermal mixing^79^,^80^ and Nuclear Overhauser Enhancement.^81^ The 60 G PTF, that gives the maximum ^13^C signal, matches the field where the NH proton of *d*_7_-BnNH_2_ becomes most strongly hyperpolarized^66^ and consequently that of the OH proton responsible for driving this change.^67^

Fig. 6e shows the level of detected SABRE-Relay polarization achieved as a function of time shaken in the PTF field. These d-glucose-^13^C_6_-*d*_7_ results come from a sample that was refilled with *p*-H_2_ after each shake. There is a clear maximum after which the signal falls. The initial increase in signal level is attributed to the continued build-up of hyperpolarization during the period of exchange with *p*-H_2_. If the *p*-H_2_ supply were to fall, there would be a drop-off in the visible signal gain due to relaxation effects and that is exactly what is illustrated in Fig. 6e. It is important to realise that even if the *p*-H_2_ supply was infinite, we would expect to reach an equilibrium point where the effects of relaxation match those of hyperpolarization build up. The concept of relaxation is, however, complicated because there are a number of effects that will take place in low field that are associated with relaxation of the *p*-H_2_ derived hydride ligands, relaxation of the bound and free NH protons, relaxation of the OH protons of glucose and relaxation of the ^13^C alignment, all of which are field dependent. These will be averaged by the time-dependent evolution of the chemical system according to the associated exchange rates and change on transfer into high field. The optimum shake time is therefore an important parameter if the maximum signal strength is required. It is 15.3 s under these conditions.

### Quantification of glucose levels by SABRE-relay

**Fig. 7:**
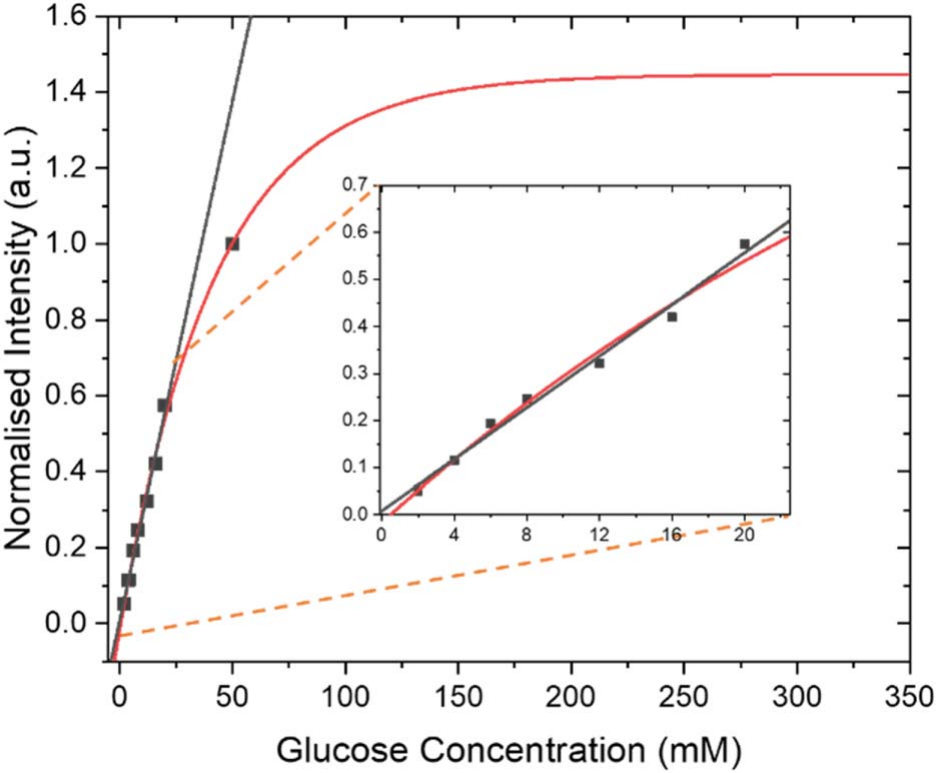
Intensity of SABRE-Relay hyperpolarized ^13^C{^2^H} signal response for d-glucose-^13^C_6_-*d*_7_ from samples containing 23.8 mM benzylamine-*d*_7_ and 4.8 mM of ***d*_22_-5** in 0.65 ml of DCM : DMF (1.6 : 1 mixture) at 9.4 T as a function of glucose concentration. In each case the sample was shaken for 10 s in a 60.6 G magnetic field. These data have been fitted to a single exponential build-up function (red line) and a linear change (black line) over the range 0–20 mM. The fitted lines have been extrapolated to show the potential theoretical signal increase with concentration. Inset shows an expansion to highlight the linear range.

In addition to the optimization steps already taken, the effect of glucose concentration on the SABRE-Relay signal was also studied. Several different glucose concentrations (d-glucose-^13^C_6_-*d*_7_) were assessed in the range 0–50 mM whilst keeping all other constituents constant, 23.8 mM *d*_7_-BnNH_2_ and 4.8 mM of ***d*_22_-5** in a DCM-*d*_2_ : DMF (1.6 : 1) mixture. The normalized results against the reference 50 mM loading are shown in Fig. 7 and detail a signal increase with increasing concentration of glucose that fits to an exponential build-up (red line) although an essentially linear response (black line) results over the range 0–20 mM. The clear linear relationship suggests that, with careful calibration, SABRE-Relay could be used to quantitatively determine the concentration of small amounts of glucose in a single scan; such an experiment would take no more than 2 minutes. This behaviour is not unexpected as Tessari *et al.* has illustrated that the SABRE response is linear at low analyte concentrations.^82^

### Glucose tautomeric analysis

All the hyperpolarization measurements shown so far yield signals for the α- and β-forms of glucose, with the C-1 resonance being clearly distinguishable. This signal provides a route to quantifying the relative amounts of the two isomers in the solution by directly comparing their hyperpolarized signals. Taking the data displayed in Fig. 6a for the ***d*_22_-5** catalyst, which involved several repeat hyperpolarization measurements, it was possible to obtain an average signal ratio. This suggested that β-glucose and α-glucose forms were present at 53.0 ± 0.2% and 47.0 ± 0.2% respectively. A thermal reference, which took 9.5 hours to acquire, gave 54.6% and 45.4% respectively (full analysis in ESI†).

Since these two numbers are comparable this suggests that the hyperpolarized measurements can be used to quantify the distribution of these isomers in solution on a timescale of a few tens of seconds. More importantly it demonstrates that SABRE-Relay is internally quantitative. The equilibrium distribution of these two isomers is 64% β-glucose and 36% α-glucose, when measured in water.^83^ The shift in ratio observed here will reflect the change in solvent.^7^

### Fructose hyperpolarization

Fructose shares a similar motif to that of glucose and should therefore polarize *via* this method. It is reported for D-DNP measurements that measured *T*_1_ values for some resonances in fructose reach up to 24 seconds.^35^ If this is the case, then it would provide significantly longer hyperpolarization lifetimes when compared to glucose. The SABRE-Relay of d-fructose-^13^C_6_-*d*_7_ was therefore undertaken. Hyperpolarized lifetimes were determined for all resonances on a sample using **3** as the pre-catalyst with 19.9 mM of d-fructose-^13^C_6_-*d*_7_ in 0.65 mL solvent (DCM-*d*_2_ : DMF (1.6 : 1)) at 9.4 T. The lifetime of the C-2 resonance was 20.3 ± 0.7 seconds which is significantly higher than the corresponding C-1 resonance of the d-glucose-^13^C_6_-*d*_7_ of 11.3 ± 0.3 seconds under the same conditions. d-Fructose differs from d-glucose by the CH_2_OH group that replaces a hydrogen in the C-2 position. Consequently, it adds a quaternary carbon centre into the molecule. Quaternary carbons typically have longer relaxation times due to the lack of directly bound ^1^H nuclei. When considering the C-1,3,4,5,6 resonances of fructose together, a hyperpolarization lifetime of 10.4 ± 0.3 seconds was determined, which is comparable to the corresponding glucose measurements (more detail in ESI†). Moreover, even in the absence of the deuterium labelling, the quaternary carbon exhibits a 12.8 ± 0.2 s hyperpolarized *T*_1_ value for the C-2 resonance, although for the other resonances it is only 2.11 ± 0.03 s (see ESI† for more detail).

When comparing the hyperpolarized NMR spectra of glucose and fructose it immediately becomes clear that there are more ^13^C resonances present in the latter. This reflects the fact that fructose exists as fructopyranose (six-membered ring) or fructofuranose (five-membered ring) in solution.

**Fig. 8:**
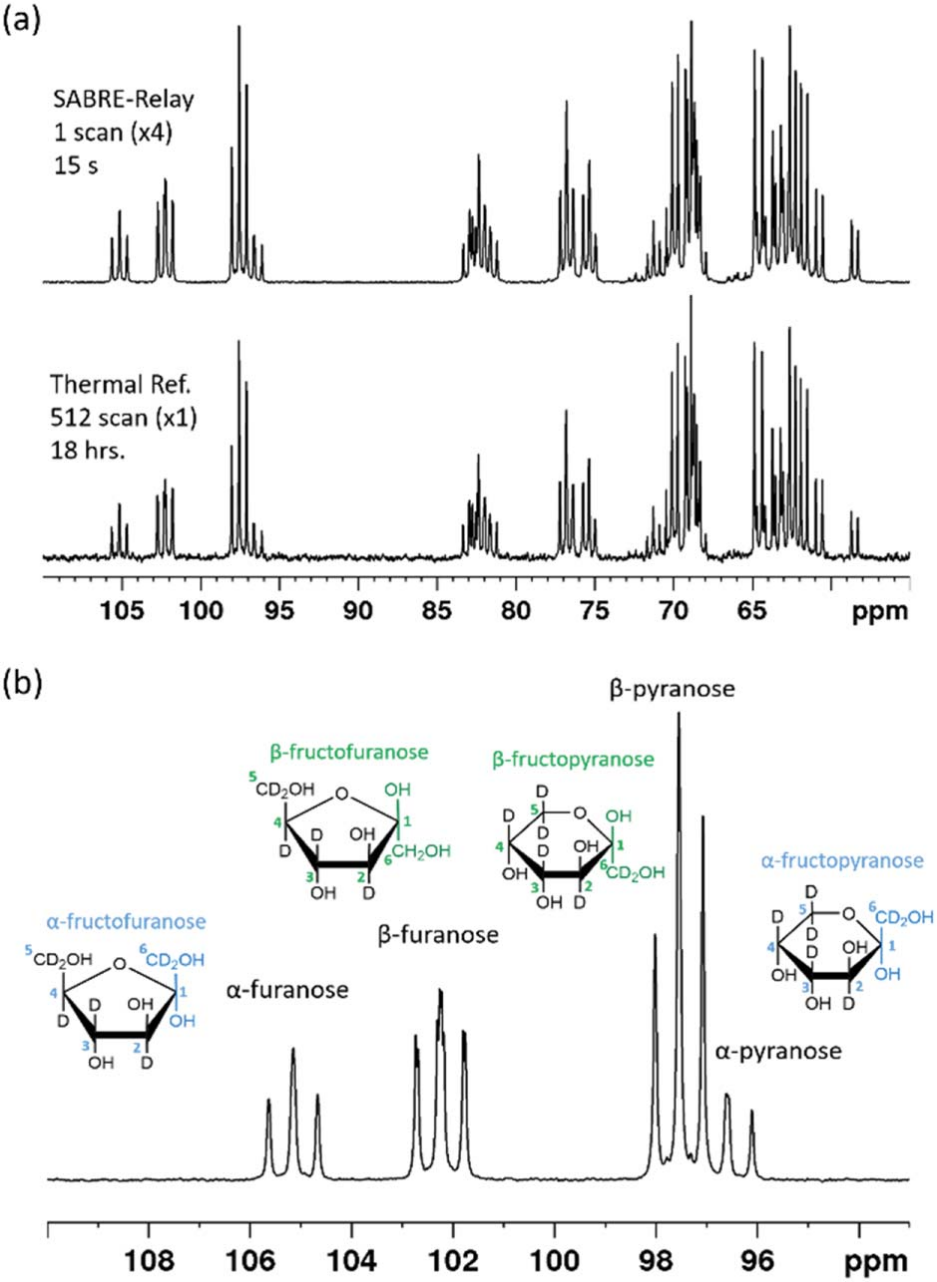
(a) SABRE-Relay hyperpolarized ^13^C{^2^H} NMR spectrum of 19.9 mM of ^13^C_6_-*d*_7_-fructose with 23.8 mM benzyl-*d*_7_-amine and 4.8 mM [IrCl(COD)(IMes)] of ***d*_22_-5** in a 0.65 ml DCM-*d*_2_ : DMF (1.6 : 1) mixture measured at 9.4 T (top spectrum) with the corresponding thermal reference, averaged over 512 scans (bottom spectrum, 18 h). (b) Expansion of (a) to highlight the different isomeric forms indicated by their C-2 resonances.

Each of these rings can exhibit α and β forms giving rise to the additional resonances observed. While glucose can exist as glucofuranose this form is typically present at <1% and is not expected to be readily observed.^8^Fig. 8a shows a hyperpolarized ^13^C{^2^H} NMR spectrum of a 19.9 mM sample of d-fructose-^13^C_6_-*d*_7_ with 23.8 mM benzylamine-*d*_7_ and 4.8 mM ***d*_22_-5** in a 0.65 ml DCM-*d*_2_ : DMF (1.6 : 1) sample measured at 9.4 T. The total enhancement factor of the measurements displayed in Fig. 8a across all of the resonances is 171 ± 2, which is of a similar order to that for glucose. The C-2 peaks of all four forms of fructose are distinct and fall in the range of 95–105 ppm. Each of these resonances has a triplet multiplicity due to the two directly bound ^13^C nuclei that couple to them. These C-2 signals appear at 104.6, 101.6, 96.9 and 95.9 ppm, and correspond to the α-d-fructofuranose, β-d-fructofuranose, β-d-fructopyranose and α-d-fructopyranose respectively (highlighted in Fig. 8b). Five repeat hyperpolarization experiments were carried out to determine an average distribution of these isomers, where each experiment takes a few minutes to complete. Their relative distributions were 15.11 ± 0.01%, 29.52 ± 0.03%, 43.83 ± 0.04% and 11.55 ± 0.03% respectively. Therefore, this result implies that the favoured form is the six-membered fructopyranose which contributes 55.38 ± 0.03%. Typically in aqueous solutions the fructopyranose form is the favoured tautomer and accounts for around 70% of the molecules with the fructofuranose being responsible for around 22%, with the remaining being other lesser forms such as the keto form.^7^,^8^ The value obtained here is obviously less than that observed in aqueous solutions, however it has been reported previously that when using ethanol : water mixtures that the fructofuranose form shifts from 27% to 60% with the change of solvent.^7^ Therefore the shift observed here is likely due to the use of the DCM-*d*_2_ : DMF mixture. A 512 scan thermally polarized spectrum was acquired over 18 hours for comparison. This yielded a distribution of 13.84%, 29.51%, 45.90% and 10.76% respectively. These proportions are again comparable to those obtained by SABRE-Relay. This highlights that hyperpolarized measurements acquired in tens of seconds can be used to accurately determine speciation in both glucose and fructose.

### Natural abundance glucose and fructose

**Fig. 9:**
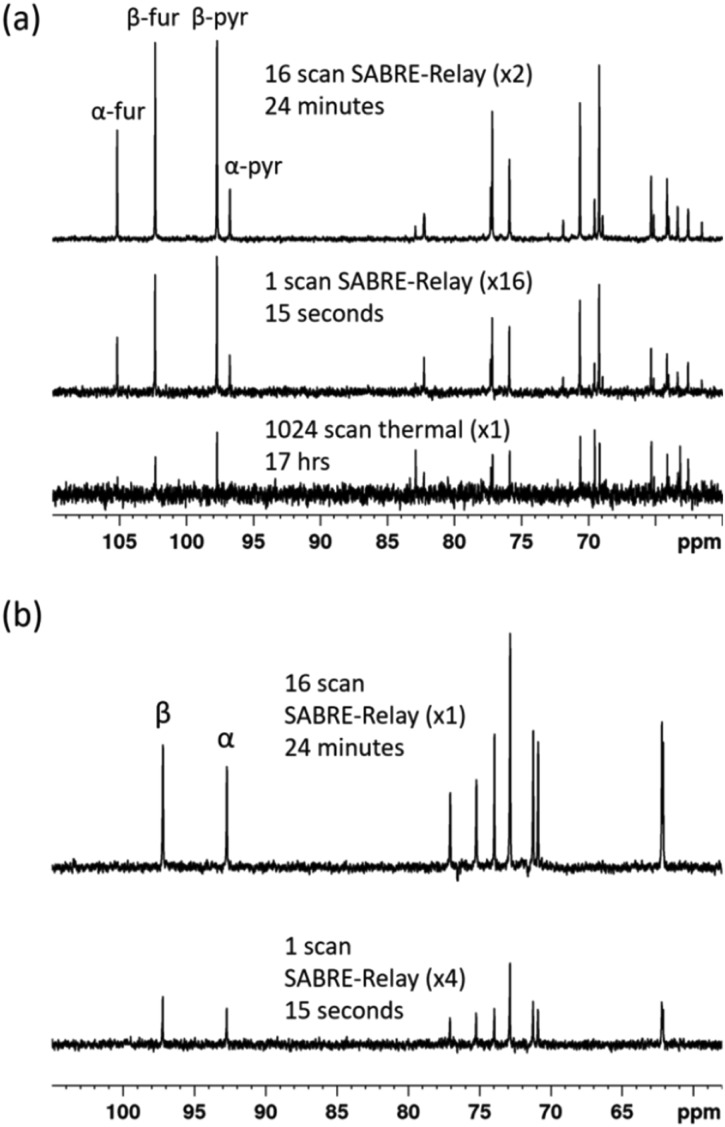
(a) ^13^C{^1^H} NMR spectra acquired for 40 mM of d-fructose (natural ^13^C abundance) with 23.8 mM benzyl-*d*_7_-amine and 4.8 mM of ***d*_22_-5** in a 0.65 ml DCM-*d*_2_ : DMF (1.6 : 1) mixture measured at 9.4 T. The bottom spectrum shows the result of signal averaging over 1024 scans (approx. 17 h), the middle spectrum represents the single scan SABRE-Relay hyperpolarization measurement and the bottom spectrum shows the 16 SABRE-Relay accumulation. (b) shows an analogous measurement for 40 mM d-glucose (natural ^13^C abundance) with 23.8 mM benzyl-*d*_7_-amine and 4.8 mM of ***d*_22_-5** in a 0.65 ml DCM-*d*_2_ : DMF (1.6 : 1) mixture measured at 9.4 T. The bottom spectrum in (b) shows a single scan hyperpolarized SABRE-Relay experiment while the top spectrum shows the result from the accumulation of 16 hyperpolarized FIDs. The vertical scaling of the three spectra was selected to establish comparable levels of noise.

Throughout this work we have used labelled compounds as they have proven to be easy to detect. However, it would be of great interest if we could achieve a detectable signal when an unlabelled sample is examined. To start this process, d-fructose (natural ^13^C abundance) was examined. The resulting ^13^C{^1^H} SABRE-Relay NMR spectrum is shown in Fig. 9a for a 40 mM loading of d-fructose (natural ^13^C abundance) with 23.8 mM benzyl-*d*_7_-amine and 4.8 mM of *d*_22_-5 in a 0.65 ml DCM-*d*_2_ : DMF (1.6 : 1) mixture that was measured at 9.4 T. Despite the vastly reduced number of ^13^C nuclei in the solution a reasonable amount of signal is obtained. Furthermore, owing to the fact that SABRE-Relay is a completely reversible technique on the addition of fresh *p*-H_2_, it is possible to take several repeat hyperpolarization 1 D experiments and to compile them into a single free induction decay (FID).

If the amount of hyperpolarization is similar in each case (there is typically just a few percent variation) then this is much the same as signal averaging in conventional NMR and would also be quantitative. The top spectrum in Fig. 9a shows the NMR spectrum resulting from the accumulation of 16 SABRE-Relay FIDs. The signal to noise increase compares well with the expected 4-fold improvement for conventional NMR. Additionally, as we are now relying on natural abundance, the additional homonuclear ^13^C couplings are no longer present which greatly simplifies the resulting spectra. The bottom spectrum of Fig. 9a serves to highlight the usefulness of this technique as this thermal reference spectrum was acquired with 1024 scans of signal averaging (approx. 17 h). The timescale of the single hyperpolarized spectrum is around 15 seconds, whereas the accumulated 16 scan experiment had a complete experimental time of around 24 minutes, although this could be reduced to under 5 minutes if used in conjunction with an automated reversible flow device that was DCM-*d*_2_ tolerant.^84^ The four isomeric forms for fructose have been determined from these data. The average response levels from these spectra yield a breakdown of 15.6 ± 0.3%, 33.1 ± 0.5%, 10.3 ± 0.6% and 41.0 ± 0.5% for the α-fructofuranose, β-fructofuranose, α-fructopyranose and β-fructopyranose respectively. The variation between these points was more significant than when observing the labelled versions as the signal to noise here is much smaller, therefore introducing more error (see ESI†). The distributions were also determined from the accumulated scan which gave 15.48%, 32.89%, 11.21% and 40.42% for the α-fructofuranose, β-fructofuranose, α-fructopyranose and β-fructopyranose respectively. These values are as expected very similar to taking an average of the individual scans, and comparable to that determined for the isotopically labelled fructose.

Analogous experiments were performed for a 40 mM natural abundance d-glucose sample, with 23.8 mM benzyl-*d*_7_-amine and 4.8 mM of ***d*_22_-5** in a 0.65 ml DCM-*d*_2_ : DMF (1.6 : 1) mixture measured at 9.4 T. The resulting spectra are shown in Fig. 9b where the bottom spectrum is a single hyperpolarized SABRE-Relay experiment and the top spectrum shows the accumulation of 16 hyperpolarized FIDs. Similarly, the glucose signal to noise is increased when the hyperpolarized scans are signal averaged. Based on analysis of the C-1 resonance, the average amounts of α-glucose and β-glucose were found to be 43 ± 1% and 57 ± 1% respectively. The values from the accumulated scan were 44.7% and 55.3% respectively, comparable to the isotopically labelled data. It can therefore be concluded that diagnostic natural abundance ^13^C NMR spectra of glucose and fructose can be measured in a single scan taking just 15 seconds that also reveal an estimate of the isomer distribution; the accuracy of this data can be improved by signal averaging.

## Conclusions

In this work we report on an in-depth study using the *para*-hydrogen based hyperpolarization technique SABRE-Relay to examine the ^13^C-labelled monosaccharides glucose and fructose. This method relies on exchange between the hyperpolarized NH protons of benzyl-*d*_7_-amine and the OH functional groups of glucose or fructose. We have shown that the resulting signal enhancements provide for the ready detection of their unambiguous fingerprint *via*^13^C NMR spectroscopy.

The relaxation lifetimes of the ^13^C nuclei in their CH groups have been studied and shown to be relatively short at around 2 seconds for both glucose and fructose at both 9.4 and 1 T. Two ways to improve on these values have been exemplified that involve deuteration or RF singlet state formation. The lifetimes of the spin state increase to 11.8 s and 9.9 s using the deuteration and singlet state techniques, respectively. This result implies that if a ^13^C_2_ labelled version of glucose or fructose (glucose-1,2-^13^C_2_ or fructose-2,3-^13^C_2_) were employed with additional ^2^H labelling on the ^13^C-sites then the corresponding singlet would exist with a lifetime that exceeds one minute. However, such selectively isotopically labelled sugars are difficult to synthesize and cannot currently be sourced commercially.

Through optimization of the catalytic system, the maximum signal enhancements observed here for the reported ^13^C measurements are on the order of 250-fold at 9.4 T (0.2% polarization). This enhancement is sufficient to enable the rapid quantification of the distribution between isomeric forms of both glucose and fructose. For example, the four distinct isomers for fructose proved to be present at 15.11 ± 0.01%, 29.52 ± 0.03%, 43.83 ± 0.04% and 11.55 ± 0.03% for the α-d-fructofuranose, β-d-fructofuranose, β-d-fructopyranose and α-d-fructopyranose tautomers respectively in the corresponding 2.5 mg sample. These values were comparable to those determined by conventional NMR spectroscopy over a timescale of 18 hours. However, the timescale for SABRE-Relay detection was just 15 seconds and hence we predict it reflects a valuable and rapid technique by which to determine such a distribution. For glucose the corresponding distribution determined in a single hyperpolarization step was α-glucose 47.0 ± 0.2% and β-glucose 53.0 ± 0.2%. This matches with the thermal reference distribution of 45.4% and 54.6% for the α-glucose and β-glucose which again to 18 h to undertake. For these types of molecule it has been demonstrated that a linear response of the SABRE-Relay derived signal integrals with concentration can be collected over the range 0–20 mM. Hence this provides a potential route to quantitation of these molecules in low concentration solutions.

Consequently, we have established that the distribution of hyperpolarization achieved by SABRE-Relay amongst the different isomers is internally quantitative whilst yielding a precise measure of amount. These measurements were extended to non-isotopically labelled variants to ensure their use in these specialist applications is possible. While, the signal to noise is significantly lower, the rapid and reliable determination of the distribution of isomers is still possible in a single scan. To gain further accuracy in these cases it has been shown that it is also possible to exploit the inherent reversibility of SABRE-Relay to signal average thereby overcoming the reduced signal to noise.

We are confident that the result presented here will have significant future impact on the analysis of sugars and suggest that with further optimization use in metabolic MRI may become possible. We are now seeking to further increase the hyperpolarization levels through the use of high *para*-hydrogen pressures and further catalyst optimization.

## Abbreviations

SABRE: Signal amplification by reversible exchange
NMR: Nuclear magnetic resonance
*p*-H_2_: *Para*-hydrogen
PTF: Polarization transfer field
NHC: N-Heterocyclic carbene
COD: 1,5-Cyclooctadiene
RF: Radio frequency
PFG: Pulse field gradient
LLS: Long lived state

## Author contributions

Initial saccharide polarization method, initial optimization and early qualitative measurements using labelled and unlabelled materials WI; development of quantitative approaches, rigorous isomeric analysis and assignments, extensive optimization, *T*_1_ assessments, benchtop NMR measurements and extension to natural abundance PMR; data modelling to theory and experimental refinement PMR, MEH, SBD; singlet lead SSR; catalyst synthesis and design PJR; research concepts WI, PMR, SBD; manuscript draft PMR (with relevant input from PJR and SSR); manuscript revision PMR, SBD; final edit: PMR, SBD, SSR, PJR and MEH.

## Funding sources

Financial support from the Wellcome Trust (Grants 092506 and 098335), the MRC (MR/M008991/1) and the EPSRC (Impact Accelerator Award G0025101 and EP/M020983/1) is gratefully acknowledged.

## Conflicts of interest

There are no conflicts to declare.

## Supplementary Material

Supplementary information
