# Supplementary material for: Rapid 13C NMR hyperpolarization delivered from para-hydrogen enables the low concentration detection and quantification of sugars
Source: Chem Sci. 2019 Sep 24;10(45):10607–19. doi: 10.1039/c9sc03450a (PMC7020793; doi:10.1039/c9sc03450a)
Supplement: Supplementary file 1 [file SC-010-C9SC03450A-s001.pdf]

Electronic Supplementary Material (ESI) for Chemical Science.
This journal is © The Royal Society of Chemistry 2019

Rapid 13C NMR Hyperpolarization delivered from para-hydrogen enables the low concentration detection and quantification of sugars

Peter M. Richardson, Wissam Iali, Soumya S. Roy, Peter J. Rayner, Meghan E. Halse and Simon B. Duckett*

Centre for Hyperpolarisation in Magnetic Resonance, Department of Chemistry, University of York, UK
KEYWORDS Hyperpolarization, para-hydrogen, glucose, fructose, SABRE, SABRE-Relay

Contents

Glucose Peak Assignment S2

Fructose Peak Assignment S3

Enhancement Value Calculation S5

Single-Shot Hyperpolarized *r.f* pulse Angles S6

Thermal T_1 and Hyperpolarized T_1 Comparison S7

Measuring the thermal T_1 with and without catalyst S10

Catalyst Effect on Hyperpolarized T_1 Values S13

Fructose Lifetime Measurements with ^{2}H labelling S15

Fructose Lifetime Measurements without ^{2}H labelling S18

SABRE-Relay Shake Time Dependence S21

Isotopic Labelled Glucose Isomer Analysis S22

Natural Abundance Glucose Isomer Analysis S23

Isotopic Labelled Fructose Isomer Analysis S25

Natural Abundance Fructose Isomer Analysis S28

References S30

Glucose Peak Assignment

In this work multiple glucose variants are used, containing different isotopic labelling combinations of both ^{13}C and ^{2}H (*d*) isotopes. In this section a concentrated naturally abundant ^{13}C sample was used to assign the peaks in glucose. The motive behind using a naturally ^{13}C-abundant sample is to remove the homonuclear ^{13}C couplings that would be present if multiple ^{13}C labels are present. The sample contained 1 M D-glucose, in D_2O. A thermal reference spectrum was taken with a total of 3600 scans in an experiment time of around 60 hours. The insets of Figure S1 show zoomed regions the spectrum which have been colour coded for ease. Also, the structures of the two isomeric forms of the glucose molecule have been added and numbered. The corresponding peak labels have also been colour coded with the blue and green labels corresponding to the α and β forms respectively.
In the spectrum shown in Figure S1 there are no observable couplings as decoupling of ^{1}H yields a singlet for each ^{13}C resonance.

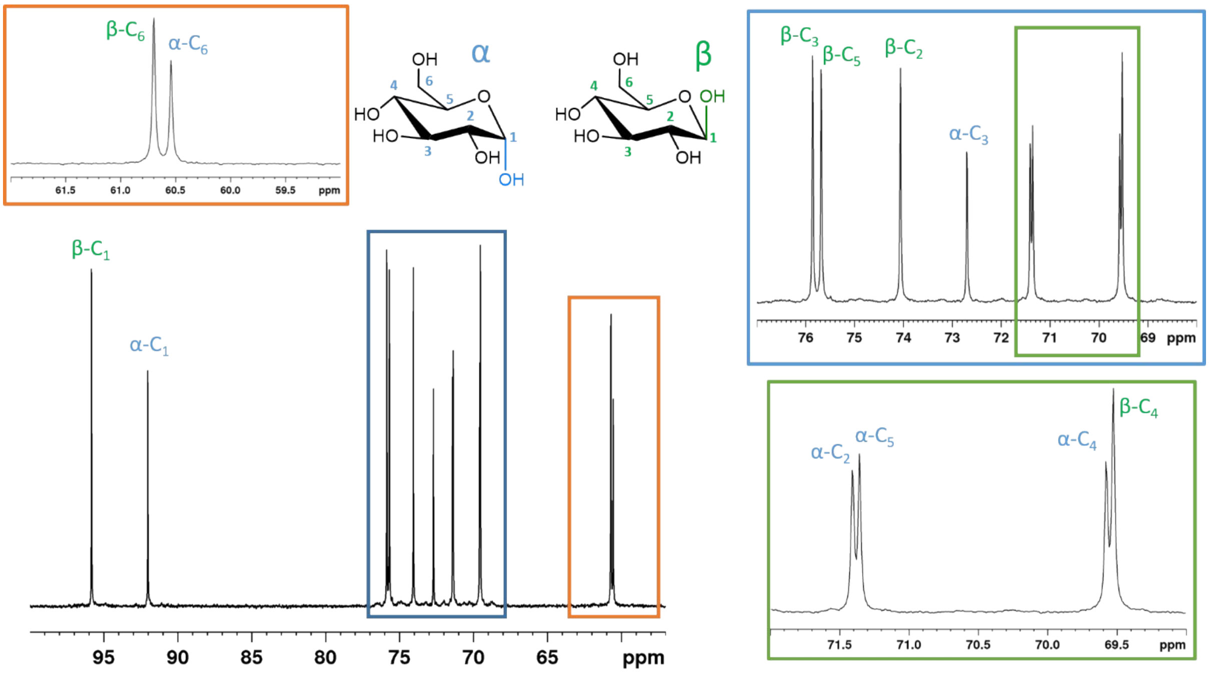

Figure S1 – A thermal reference scan taken for a 1 M D-Glucose sample in D_2O acquired on a 9.4 T NMR spectrometer with 3600 scans (60 hrs). The insets are colour coded with zoomed regions of the spectrum. The structure of glucose has been given in both of its isomeric forms α and β and the subsequent labels have been colour coded.

The ^{13}C chemical shifts of the glucose resonances are well known, the peaks in Figure S1 having been assigned based on chemical shift information taken from previously the work of Pfeffer *et al.*[1] The values from the work of Pfeffer *et al* are shown in Table S1 showing resonances for both the α-D-Glucose and β-D-Glucose isomers.

Table S1 – ^{13}C Chemical shift assignments for α-D-Glucose and β-D-Glucose taken from published work by Pfeffer *et al.*[1]

	13C Chemical Shift (ppm)					
	C-1	C-2	C-3	C-4	C-5	C-6
α-D-Glucose	92.94	72.47	73.75	70.56	72.28	61.59
β-D-Glucose	96.74	75.14	76.71	70.6	76.78	61.74

Fructose Peak Assignment

A concentrated naturally abundant ^{13}C sample of D-Fructose was also analysed. This sample contained 0.58 M D-Fructose in a mixture of solvent DCM-d2: DMF (1:0.75) to aid in dissolution of the molecule. In the previous section the glucose was determined using D_20 as the solvent, this was not used here as one of the isomeric forms observed in the hyperpolarized spectrum does not occur in water. Therefore, the same mix of solvents as used for the hyperpolarized measurements was used here to provide a direct comparison. A thermal reference spectrum was taken with a total of 3700 scans over an experiment time of around 67 hours. The insets of Figure S2 shows zoomed in regions which have been colour coded for ease. Also, the structures of the four isomeric forms of the fructose molecule have been added and numbered. The corresponding peak labels have also been colour coded with the blue and green labels corresponding to the α and β forms respectively.
As in the previous section it should be noted that in the spectrum shown in Figure S2 there are no observable couplings due to decoupling.

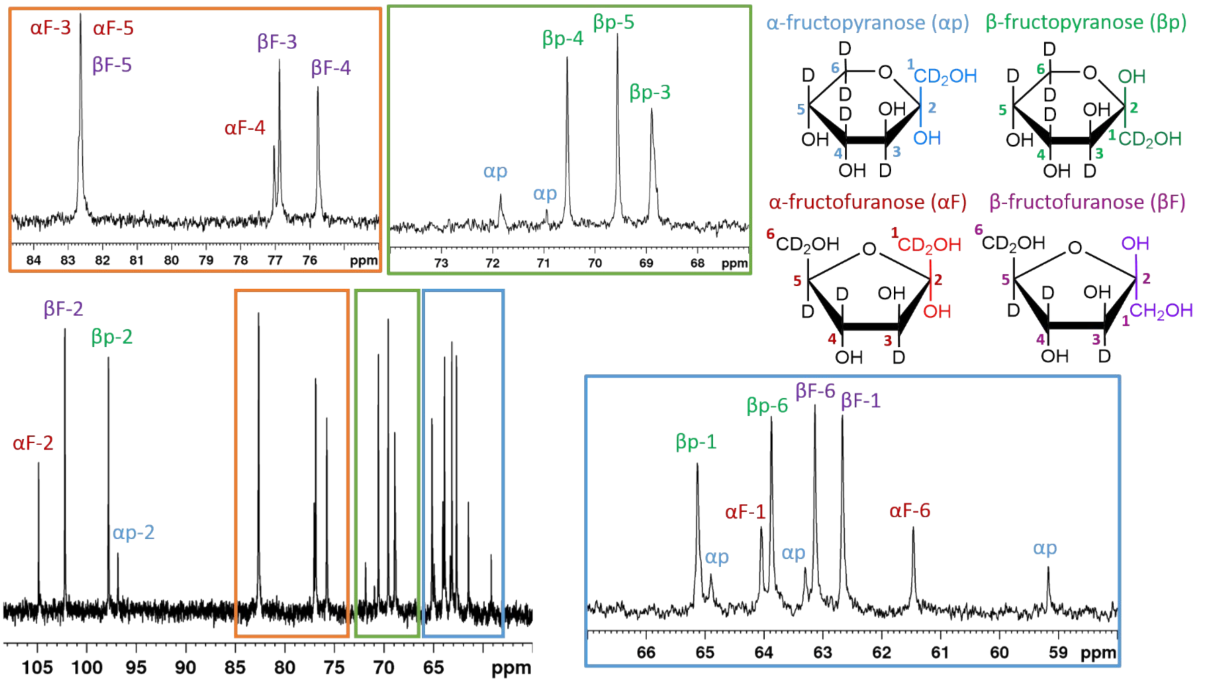

Figure S2 – A thermal reference scan taken for a 0.58 M D-Fructose sample in D_2O acquired on a 9.4 T NMR spectrometer with 3700 scans (67 hrs). The insets are colour coded with zoomed regions of the spectrum. The structure of fructose in all four present isomers has been given with subsequent labels which have been colour coded. The αF-3, αF-5 and βF-5 peak all overlap around 82.5 ppm, as shown in inset.

The chemical shift information of D-Fructose is already well established, subsequently the peaks in Figure S2 have been assigned using the chemical shift information of published work by Pfeffer *et al*.[1] In Figure S2 it can be seen that the resonances have been assigned for the β-D-fructopyranose, α-D-fructofuranose and β-D-fructofuranose isomers but not for the α-D-fructopyranose form. This is because typically this is only formed in very small quantities and not commonly characterized for this reason. However, it is clear that the resonance at 96.86 ppm is responsible for the C-2 resonance of the α-D-fructopyranose form. In the discussions within this work only the C-2 resonances are used in the analysis of the isomeric distributions as they are distinct from the other carbon resonances.

Table S2 – ^{13}C Chemical shift assignments for β-D-fructopyranose, α-D-fructofuranose and β-D-fructofuranose taken from published work by Pfeffer *et al*.[1] In the experiments carried out here we also note that the α-D-fructopyranose form can be seen, however this isomer is usually present in negligible amounts so is less characterized.

	13C Chemical Shift (ppm)					
	C-1	C-2	C-3	C-4	C-5	C-6
β-D-fructopyranose	64.91	98.89	68.57	70.68	70.16	64.24
α-D-fructofuranose	63.94	105.23	82.96	77.02	82.16	62.08
β-D-fructofuranose	63.71	102.31	76.37	75.41	81.51	63.34

Enhancement Value Calculation

The enhancement factors quoted in the hyperpolarised data were determined by taking a ratio of the integrals of the relevant peak in the hyperpolarized NMR spectrum and the thermally polarized NMR spectrum according to equation S1.

$$\epsilon = \frac{\text{integral of hyperpolarized NMR spectrum}}{\text{integral of thermal NMR spectrum}} n \qquad \text{(S1)}$$

Where n is the number of scans used to obtain the thermal reference spectrum. Since in this paper we are undertaking ^{13}C hyperpolarization, obtaining a thermal reference scan requires a lot of signal averaging and thus high experimental times. Typically for the isotopically labelled samples at the concentrations used in this study (around 20 mM) 512 scans were performed. As such, for each individual study such as amine type, glucose concentration, catalyst type and shake time a single thermal reference was taken in each instance to calculate the enhancement factors, as in each case the solutions were made up from bulk solutions.

Single-Shot Hyperpolarized *r.f* pulse Angles

The single-shot sequence to determine the hyperpolarized lifetime of the target analytes have proven a useful and time efficient. The method involves a single hyperpolarization step, followed by insertion into the magnet and commencement of the sequence. The sequence employs multiple pulses which are spaced by a user inputted time, where each of the pulses has a longer duration than the last. The aim of the sequence is to provide the same proportion of magnetization after each pulse, where the pulse duration increases to account for the loss in residual magnetization due to excitation. The pulse angles below were used and are optimised for a 15 point sequence. If more points are required then less magnetization can be sampled each time; there is a trade-off with signal to noise. The maximum amount of signal obtained for this setup is 25.8 % of the total possible provided by the SABRE-Relay result.

Table S3: Pulse angles and durations used for the single-shot hyperpolarized lifetime measurements, showing that the same amount of magnetization is taken each time and the remaining magnetization after each step. The pulse durations were calculated on the basis that there would be 15 points acquired.

n	M_{xy} (%)	$M_{z,n}$ (%)	Pulse Angle (°)	Pulse Duration (µs)
1	25.8	96.6	15.0	2.49
2	25.8	93.1	15.5	2.58
3	25.8	89.4	16.1	2.68
4	25.8	85.6	16.8	2.80
5	25.8	81.6	17.6	2.93
6	25.8	77.5	18.4	3.07
7	25.8	73.0	19.5	3.25
8	25.8	68.3	20.7	3.45
9	25.8	63.2	22.2	3.70
10	25.8	57.7	24.1	4.02
11	25.8	51.6	26.6	4.43
12	25.8	44.7	30.0	5.00
13	25.8	36.5	35.3	5.88
14	25.8	25.8	45.1	7.51
15	25.8	0.0	90.0	15.00

Table S3 shows the pulse angles needed for maximum optimised signal for a 15-point single-shot experiment. After each acquisition the change in the M_{xy} (magnetization in the xy-plane) and M_z (magnetization in the z-plane) is as follows where $M_{z,0}$ is normalized to 1:

$$M_{xy,n} = M_{z,n-1} sin\theta \; and \; M_z = M_{z,n-1} cos\theta \quad (S2)$$

Thermal T_1 and Hyperpolarized T_1 Comparison

In the main text we make the assumption that since hyperpolarization is a manipulation of spin states and thus magnetization, the rate of relaxation of such magnetization should be independent of the intensity of the starting signal. Therefore, we assume that the hyperpolarized lifetimes measured are comparable to that of thermal NMR. In this section we experimentally prove this hypothesis.

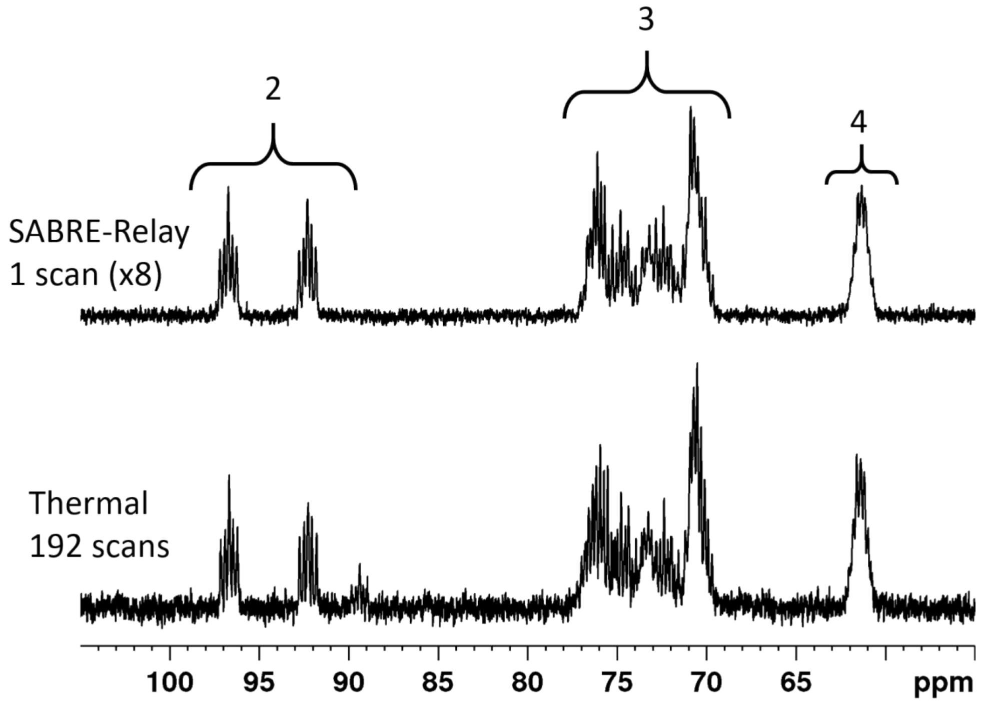

Figure S3 – Thermal and hyperpolarized T_1 values have been determined for a sample containing 20 mM D-Glucose-$^{13}C_6$-d_7 with 4.8 mM of catalyst **3** and 26 mM benzyl-d_7-amine in DCM-d2: DMF (1.6: 1). The top spectrum is the first spectrum from the single-shot hyperpolarized lifetime sequence which was optimized for 15 points with 25.8 % of the maximum possible signal taken with each spectrum. The bottom spectrum is the last spectrum from a thermal reference inversion recovery sequence acquired with 192 scans. The T_1 has been determined for several spectral regions corresponding to different sites of the glucose labelled 1-4, where region 1 is all of the glucose peaks integrated together. The hyperpolarized sample was shaken for 10 seconds at 60 G prior to being inserted into the spectrometer where the single-shot sequence was initiated. Both measurements were taken on a 400 MHz spectrometer.

Thermal and hyperpolarized T_1 values have been determined for a sample containing 20 mM D-Glucose-$^{13}C_6$-d_7 with 4.8 mM of catalyst **3** and 26 mM benzyl-d_7-amine in DCM-d2: DMF (1.6: 1). The top spectrum in Figure S3 is the first spectrum from the single-shot hyperpolarized lifetime sequence which corresponds to 25.8 % of the maximum possible signal. An inversion recovery was used to measure the thermal T_1 of the glucose and as such the last spectrum is shown in Figure S3 (bottom spectrum) as this presented the largest signal (which is expected from an inversion recovery sequence). The T_1 has been determined for several spectral regions corresponding to different sites of the glucose labelled 1-4, where region 1 is all of the glucose peaks integrated together. The hyperpolarized sample was shaken for 10 seconds at 60 G prior to being inserted into the spectrometer where the single-shot sequence was initiated. For completeness the data for each normalized integration region is shown for the thermal inversion recovery and hyperpolarized single-shot sequence in tables S3 and S4 respectively. The data in Table S3 for the inversion recovery thermal has been normalized to the last point and the inverse has been done for the hyperpolarized sequence, to reflect the highest signal in each sequence.

Table S3 – Data acquired from an inversion recovery experiment on 20 mM D-Glucose-$^{13}C_6$-d_7 with 4.8 mM of catalyst **3** and 26 mM benzyl-d_7-amine in DCM-d2: DMF (1.6: 1). A total of 192 scans were acquired for each point of the sequence with a relaxation delay of 70 seconds to allow for relaxation of all magnetization before subsequent acquisition. The data has been normalized relative to the last point in each case as this gives the largest signal in an inversion recovery sequence.

	Normalised Integral (a.u.)			
Time (s)	1	2	5	6
0.001	-0.762	-0.809	-0.785	-0.737
0.5	-0.657	-0.713	-0.697	-0.591
1	-0.565	-0.617	-0.601	-0.506
2	-0.413	-0.464	-0.469	-0.295
4	-0.151	-0.225	-0.211	-0.024
6	0.010	-0.059	-0.026	0.127
8	0.128	0.028	0.123	0.289
10	0.278	0.188	0.266	0.447
12	0.402	0.358	0.392	0.536
16	0.597	0.540	0.577	0.674
20	0.720	0.688	0.707	0.782
25	0.830	0.743	0.816	0.901
40	0.964	0.957	0.964	0.973
64	1.000	1.000	1.000	1.000

Table S4 – Data acquired from a hyperpolarized single-shot experiment on 20 mM D-Glucose-$^{13}C_6$-d_7 with 4.8 mM of catalyst **3** and 26 mM benzyl-d_7-amine in DCM-d2: DMF (1.6: 1) to determine the hyperpolarized T_1. The data represents a single scan per point using increasing angles to obtain the same magnetization at every acquisition, therefore any decrease in signal is due to relaxation only. A single hyperpolarization step preceded acquisition in the form of loading 4 bar (absolute) *para*-hydrogen into the NMR tube and shaking for 10 seconds in a magnetic field of 60 G before transferring to the magnet for detection.

	Normalised Integral (a.u.)			
Time (s)	Peak 1	Peak 2	Peak 3	Peak 4
0	1.000	1.000	1.000	1.000
4	0.757	0.812	0.758	0.674
8	0.513	0.569	0.521	0.445
12	0.354	0.370	0.373	0.286
16	0.241	0.276	0.267	0.148
20	0.158	0.176	0.173	0.125
24	0.121	0.112	0.143	0.079
28	0.067	0.078	0.091	0.039
32	0.065	0.090	0.077	0.033
36	0.037	0.027	0.053	0.034
40	0.029	0.050	0.044	-0.004
44	0.027	0.029	0.037	0.001
48	0.024	0.044	0.033	0.011
52	0.021	0.034	0.033	0.022
56	0.018	0.016	0.038	0.004

The timescale of these two experiments were around 63 hours and 70 seconds for the thermal inversion recovery and single-shot hyperpolarized sequences respectively. Two different equations were used to fit the data in Tables S3 and S4 as the behaviour of the signal is different in both cases. For an inversion recovery sequence, the magnetization spans from $-M_0$ at time zero up to $+M_0$ at very long times, where M_0 is the magnetization derived from the polarization along the z-axis. Therefore, the equation which was fitted to the data in Table S3 had the form of equation S3.

$$M_{xy} = M_0\left(1 - 2Ae^{-\frac{t}{T_1}}\right) \qquad (S3)$$

Where the parameter A has been inserted to account for any experimental imperfections but should have a value of 1 in an ideal case. The equation which was used for the determination of the hyperpolarized T_1 is a simple single exponential function in the form of equation S4.

$$M_{xy} = M_0e^{-\frac{t}{T_1}} + M_{offset} \qquad (S4)$$

Where in this case M_0 is 25.8 % of the total SABRE-Relay generated magnetization (as a 15° pulse was used for the single-shot sequence) and the parameter M_{offset} has been added to the fit in cases where the signal does not completely tend to zero, which in this case would be a measure of the noise. Example data for the hyperpolarized single-shot sequence has been shown in Table S4, however as they only take 70 seconds to acquire three repeat measurements were carried out. All resulting T_1 values are displayed in Table S5 with the relevant fitting error determined from the quality of the fit.

Table S5 – T_1 values determined from the data in Tables S3 and S4 along with two other repeat hyperpolarization experiments (data is not shown here). The three hyperpolarized values are displayed along with an average value. Each value has an error associated which was determined from the fit, with the error for the average hyperpolarization value deriving from error propagation of the individual values.

Peak #	Thermal T_1 (s)	Hyperpolarized T_1 (s)			
		1	2	3	average
1	11.2 ± 0.4	11.3 ± 0.4	11.7 ± 0.4	11.0 ± 0.3	11.3 ± 0.2
2	12.2 ± 0.5	12.1 ± 0.8	12.7 ± 0.9	12.0 ± 0.5	12.2 ± 0.5
3	11.3 ± 0.2	11.6 ± 0.4	11.8 ± 0.4	11.4 ± 0.3	11.5 ± 0.2
4	8.9 ± 0.4	9.4 ± 0.3	8.9 ± 0.2	8.7 ± 0.3	9.0 ± 0.2

From the data in Table S5 it can be clearly ascertained that the average hyperpolarized values for every integration region are very similar to the corresponding T_1 value. Therefore, this supports the hypothesis that the hyperpolarized lifetimes measurements are equivalent to measuring the thermal T_1 of the system, however the former requires 0.03 % of the experiment time of the thermal reference equivalent, which represents a significant reduction in experiment time.

Measuring the thermal T_1 with and without catalyst

Typically, in the classical SABRE approach the T_1 of the target molecule bound to the catalyst is significantly shorter than the same molecule in free solution. Therefore, the resulting observed T_1 is a weighted average of the two environments which is dependent on the concentration of the target molecule, as the concentration is increased there will be a greater proportion of molecules free in solution and thus a higher relaxation time. In the case of SABRE-Relay the target molecule does not bind to the catalyst, and as such the presence of the catalyst would have only a minimal effect on the target molecule via dipole-dipole interactions in solution. In this section we aim to show the effect of having the catalyst present and absent to observe any difference in the T_1 value.

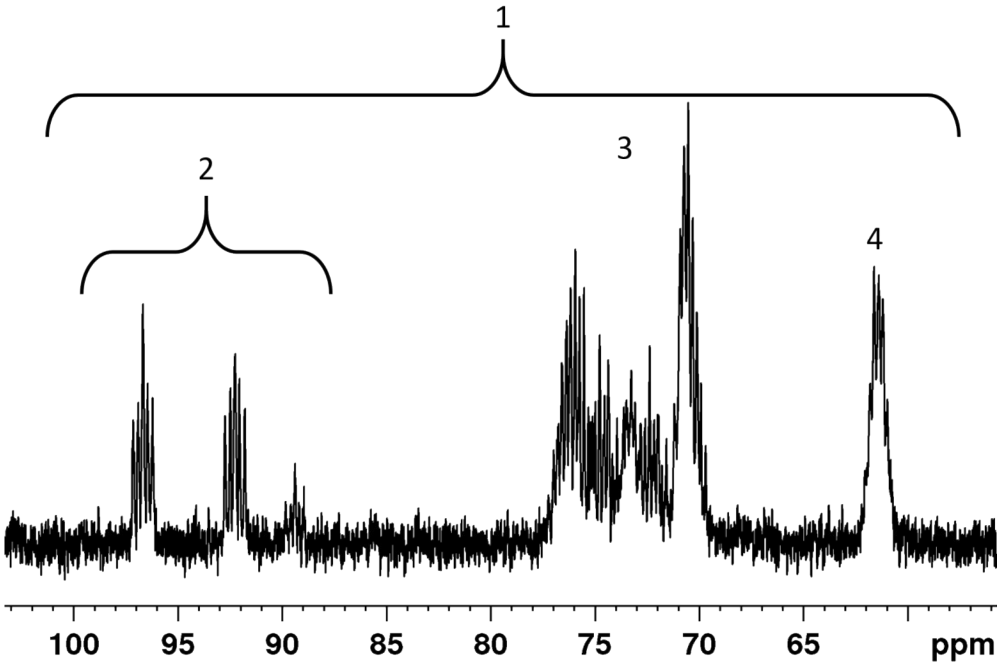

Figure S4 – Thermal spectrum of a sample containing 20 mM D-Glucose-$^{13}C_6$-d_7 with 4.8 mM of catalyst **3** and 26 mM benzyl-d_7-amine in DCM-d2: DMF (1.6: 1). The T_1 has been determined for several spectral regions corresponding to different sites of the glucose labelled 1-4, where region 1 is all of the glucose peaks integrated together.

In order to observe the effect of the catalyst presence on the T_1 value an inversion recovery measurement was conducted on two samples both containing 20 mM of D-Glucose-$^{13}C_6$-d_7, 26 mM of benzyl-d_7-amine in DCM-d_2: DMF (1.6:1), and one of the samples also contained 4.8 mM of catalyst **3**, which is the only difference between the two samples. An inversion recovery sequence was used for both experiments each with 192 scans per point with a total experiment time of 63 hours. The normalized integral data for the sample with and without catalyst is displayed in Tables S6 and S7 respectively. The same four regions have been analysed as the previous section.

Table S6 – Data acquired from an inversion recovery experiment on 20 mM D-Glucose-$^{13}C_6$-d_7 with 4.8 mM of catalyst **3** and 26 mM benzyl-d_7-amine in DCM-d2: DMF (1.6: 1). A total of 192 scans were acquired for each point of the sequence with a relaxation delay of 70 seconds to allow for relaxation of all magnetization before subsequent acquisition. The data has been normalized relative to the last point in each case as this gives the largest signal in an inversion recovery sequence. Measured on a 9.4 T (400 MHz) spectrometer with a total experiment time of around 63 hours.

	Normalised Integral (a.u.)			
Time (s)	Peak 1	Peak 2	Peak 3	Peak 4
0.001	-0.762	-0.809	-0.785	-0.737
0.5	-0.657	-0.713	-0.697	-0.591
1	-0.565	-0.617	-0.601	-0.506
2	-0.413	-0.464	-0.469	-0.295
4	-0.151	-0.225	-0.211	-0.024
6	0.010	-0.059	-0.026	0.127
8	0.128	0.028	0.123	0.289
10	0.278	0.188	0.266	0.447
12	0.402	0.358	0.392	0.536
16	0.597	0.540	0.577	0.674
20	0.720	0.688	0.707	0.782
25	0.830	0.743	0.816	0.901
40	0.964	0.957	0.964	0.973
64	1.000	1.000	1.000	1.000

Table S7 – Data acquired from an inversion recovery experiment on 20 mM D-Glucose-$^{13}C_6$-d_7 with 26 mM benzyl-d_7-amine in DCM-d2: DMF (1.6: 1). The catalyst was absent from this sample, however, all other constituents were left unchanged as to directly compare the effect of the catalyst presence. A total of 192 scans were acquired for each point of the sequence with a relaxation delay of 70 seconds to allow for relaxation of all magnetization before subsequent acquisition. The data has been normalized relative to the last point in each case as this gives the largest signal in an inversion recovery sequence. Measured on a 9.4 T (400 MHz) spectrometer with a total experiment time of around 63 hours.

	Normalized Integral (a.u.)			
Time (s)	Peak 1	Peak 2	Peak 3	Peak 4
0.001	-0.758	-0.829	-0.759	-0.695
0.5	-0.656	-0.671	-0.683	-0.609
1	-0.568	-0.621	-0.592	-0.473
2	-0.401	-0.421	-0.448	-0.290
4	-0.133	-0.094	-0.189	-0.054
6	0.020	-0.022	0.004	0.150
8	0.143	0.147	0.139	0.283
10	0.295	0.242	0.293	0.468
12	0.415	0.325	0.422	0.543
16	0.621	0.614	0.600	0.724
20	0.734	0.704	0.730	0.822
25	0.870	0.836	0.861	0.883
40	0.967	0.965	0.965	0.967
64	1.000	1.000	1.000	1.000

The resulting T_1 values from these two experiments are displayed in Table S8 for each of the integration regions 1-4. Also included in the Table S8 is the difference between the sample with and without the catalyst as a percentage. As in the previous section each of these T_1 values was determined by fitting equation S4 to the data. It can be concluded from the results displayed in Table S8 that for all regions the T_1 values are slightly larger when the catalyst is present that the case when the catalyst is absent. Although the sample with the catalyst present has longer T_1 values it should be noted that these are somewhat minor shifts with an average difference of only 3.9 % which is comparable to the level of error assigned to each of these values. However, since there is a systematic difference between these two environments it is likely that there is a factor causing a slight reduction when the catalyst is absent. Firstly, this result is the converse of classic SABRE which sees a decrease in the T_1 value with the introduction of the catalyst. One possible explanation of this effect is that when the carrier amine is bound to the catalyst it will not be undergoing hydrogen exchange with the glucose molecules, therefore this could lead to slower exchange of hydrogen onto the glucose molecule and therefore slightly longer T_1 values.

Table S8 – T_1 values determined from the data in Tables S6 and S7 for a sample with and without catalyst respectively. Each value has an error associated which was determined from the fit. Also displayed in the table is a percentage difference of the T_1 without the catalyst with respect to the measurement carried out with the catalyst present.

	Thermal T_1 (s)			
	Peak 1	Peak 2	Peak 3	Peak 4
With Cat.	11.2 ± 0.4	12.2 ± 0.4	11.3 ± 0.4	8.9 ± 0.4
Without Cat.	10.9 ± 0.4	11.1 ± 0.4	10.9 ± 0.4	8.8 ± 0.4
% diff.	2.54	9.28	2.94	0.96

Catalyst Effect on Hyperpolarized T_1 Values

In the previous section it was shown that the role of the catalyst has a limited effect on the T_1 value of the target molecule (in this case glucose and fructose). This is because we are using the SABRE-Relay method of hyperpolarization which does not involve direct interaction of the target and catalyst but rather through an intermediate carrier. In this section we present the hyperpolarized T_1 values for the glucose molecule when in the presence of the range of catalysts which were employed in the optimization process in the main text. Figure S5 shows a hyperpolarized spectrum of a sample containing 20 mM of D-Glucose-$^{13}C_6$-d_7 with 26 mM benzyl-d_7-amine and 4.8 mM of catalyst **3** in DCM-d2: DMF (1.6:1). In the previous sections it is has been clear from the T_1 data that there are two distinct T_1 values for the glucose molecule. Resonances C-1,2,3,4,5 have a T_1 value of around 12 seconds and the C-6 resonance has a value slightly lower around 9 seconds. Therefore, in this section we will focus just on these two regions which are shown graphically in Figure S5.

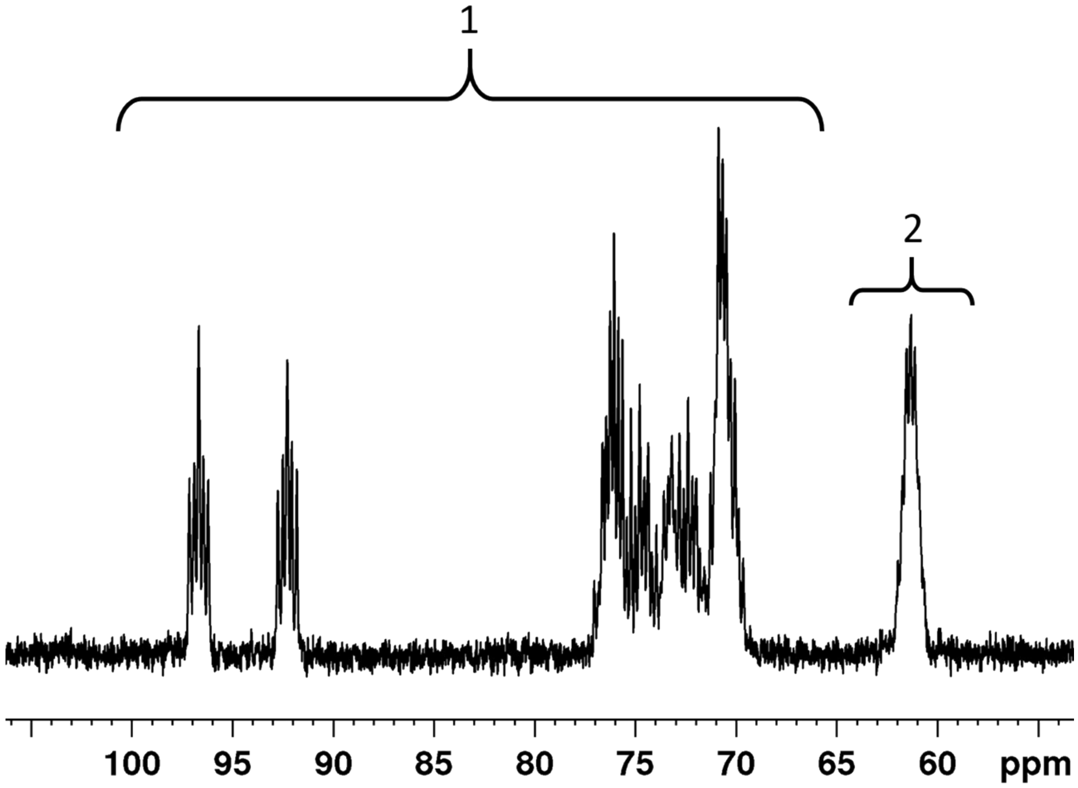

Figure S5 – Hyperpolarized spectrum of a sample containing 20 mM D-Glucose-$^{13}C_6$-d_7 with 4.8 mM of catalyst **3** and 26 mM benzyl-d_7-amine in DCM-d2: DMF (1.6: 1). Based on the previous sections which showed that there were two distinct regions with different T_1 values, two regions have been analysed here labelled 1 and 2.

The hyperpolarized T_1 values have been determined for 8 of the catalyst used in this study with various electronic and steric characteristics. The various catalysts have been shown in the main text to yield quite different SABRE-Relay enhancements. For each catalyst three repeat measurements were taken using the single-shot sequence. Before each acquisition the NMR tube was filled with fresh *para*-hydrogen at 4 bar (absolute) and shaken for 10 seconds inside a hand-held shaker held at 60 G magnetic field. The average value for the hyperpolarized T_1 for each catalyst is displayed in Table S9 where the errors have come from the fit to the data in each case and subsequently through error propagation.

Table S9 – Hyperpolarized T_1 values determined for three repeat single-shot measurements on samples containing 20 mM D-Glucose-$^{13}C_6$-d_7 with 26 mM benzyl-d_7-amine and 4.8 mM of catalyst (which is different in each case) in DCM-d_2: DMF (1.6:1). Each value has an error associated which was determined from the fit. The hyperpolarized T_1 values have been determined for the C-1,2,3,4,5 resonances together as well as for the C-6 resonance.

	Hyperpolarized T_1 (s)	
Catalyst	C-2,3,4,5	C-6
**2**	12.0 ± 0.3	9.1 ± 0.6
**3**	11.8 ± 0.3	9.2 ± 0.2
d$\boldsymbol{d_{22}}$**-3**	12.7 ± 0.3	9.3 ± 0.2
**4**	12.2 ± 0.3	9.4 ± 0.2
**5**	11.8 ± 0.1	8.9 ± 0.1
$\boldsymbol{d_{22}}$**-5**	11.2 ± 0.1	8.5 ± 0.1
**6**	11.9 ± 0.2	8.9 ± 0.2
**7**	10.7 ± 0.2	8.6 ± 0.2

The data in Table S9 shows that the hyperpolarized T_1 values are comparable in each case where the average value from all measurements comes to be 11.8 ± 0.2 seconds, where the error was determined from the standard deviation of the measurements. The data in Table S9 is shown graphically in Figure S6, along with the catalyst structures used in this work. It can be readily noted that the catalyst effect on the hyperpolarized T_1 values is negligible. This result further corroborates the theory that the SABRE-Relay method is not susceptible to the same negative effect that the classical SABRE approach falls victim.

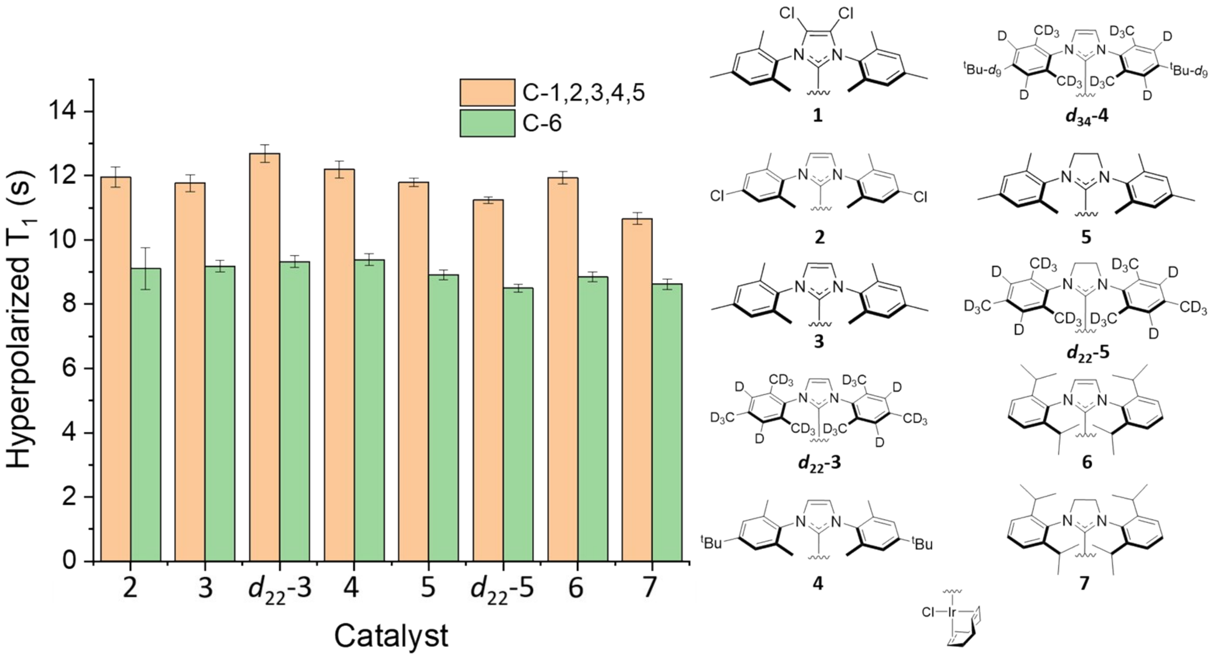

Figure S6 – Hyperpolarized T_1 values for 8 of the catalysts studied in this work for both the combined region containing C-1, 2, 3, 4, 5 and the C-6 region. The structures of the catalyst used in this research have also been displayed here for ease and the numbering has been kept the same as in the main text.

Fructose Lifetime Measurements with ^2H labelling

Fructose has been previously shown to exhibit longer T_1 values than glucose, which makes it a strong candidate to use in hyperpolarization applications. In this section we will discuss the hyperpolarized lifetimes determined for the fully deuterated D-Fructose-$^{13}C_6$-d_7. A hyperpolarized spectrum of 2.5 mg D-Fructose-$^{13}C_6$-d_7 with 4.8 mM of catalyst **3** in DCM-d_2: DMF (1.6:1) is shown in Figure S7. The Fructose measured has 24 different ^{13}C resonances from the different possible isomers. Due to the homonuclear ^{13}C coupling not all of these peaks are resolved and thus in the analysis below we have integrated as many distinguishable peaks as possible. Table S10 gives the intensity as a function of time after the hyperpolarization step, where the sample has waited inside the spectrometer. The peaks analysed are labelled 1-11 and shown in Figure S7, where peak 11 is the whole fructose region analysed in one. There are two distinct regions that are focussed on here, since the C-2 resonance is estimated to have the longest T_1, due its quaternary nature, and has a distinct chemical shift away from the other five ^{13}C resonances, the C-2 resonances resulting from all isomers of the fructose have been analysed together (peak 4) and the other resonances have also been grouped together (peak 10).

Table S10 - Intensities of the ^{13}C resonances detailed in Figure S7 for all distinguishable resonances of fructose as a function of time after the hyperpolarization step (where $t = 0$ depicts the moment after transfer to the spectrometer). This data was acquired using the manual repolarization method, in which, fresh *para*-hydrogen is added before each experiment and shaken for 10 s at 60 G. The sample contained 4.8 mM of the **3** catalyst with 2.5 mg of $^{13}C_6$-d_7-Fructose in 0.65 ml solvent (DCM:DMF (1.6:1)).

Time (s)	Normalised Intensity (a.u.)										
	**1**	**2**	**3**	**4**	**5**	**6**	**7**	**8**	**9**	**10**	**11**
0	1.000	1.000	1.000	1.000	1.000	1.000	1.000	1.000	1.000	1.000	1.000
2	0.901	0.932	0.937	0.930	0.794	0.812	0.815	0.746	0.742	0.783	0.809
4	0.802	0.834	0.832	0.827	0.661	0.659	0.669	0.587	0.583	0.633	0.667
6	0.772	0.781	0.802	0.786	0.599	0.590	0.604	0.513	0.492	0.563	0.602
8	0.668	0.710	0.717	0.706	0.507	0.493	0.500	0.418	0.414	0.465	0.508
10	0.665	0.685	0.682	0.678	0.473	0.440	0.449	0.363	0.371	0.415	0.461
12	0.542	0.558	0.559	0.552	0.355	0.319	0.331	0.257	0.248	0.301	0.345
15	0.465	0.472	0.479	0.473	0.268	0.238	0.243	0.176	0.186	0.217	0.262
20	0.363	0.377	0.368	0.367	0.174	0.150	0.157	0.112	0.126	0.139	0.180
25	0.273	0.295	0.308	0.299	0.134	0.105	0.116	0.075	0.066	0.099	0.134
30	0.227	0.250	0.237	0.238	0.090	0.070	0.078	0.048	0.023	0.065	0.096
35	0.180	0.186	0.195	0.187	0.066	0.052	0.054	0.028	0.019	0.043	0.070
40	0.147	0.139	0.143	0.140	0.041	0.030	0.036	0.017	-0.033	0.027	0.047
45	0.117	0.134	0.127	0.126	0.030	0.021	0.026	0.013	-0.025	0.019	0.039
50	0.117	0.134	0.127	0.126	0.029	0.020	0.026	0.014	-0.017	0.019	0.039
60	0.091	0.082	0.077	0.078	0.015	0.012	0.011	0.007	-0.004	0.009	0.021
70	0.032	0.041	0.054	0.044	0.006	0.006	0.007	0.006	-0.024	0.006	0.013
85	0.011	0.029	0.028	0.026	0.000	0.002	0.004	0.006	-0.016	0.003	0.009
100	0.002	0.009	0.021	0.015	-0.003	0.005	0.003	0.002	0.008	0.002	0.006
120	0.008	0.011	0.014	0.007	0.005	0.002	0.002	0.006	-0.023	0.004	0.008

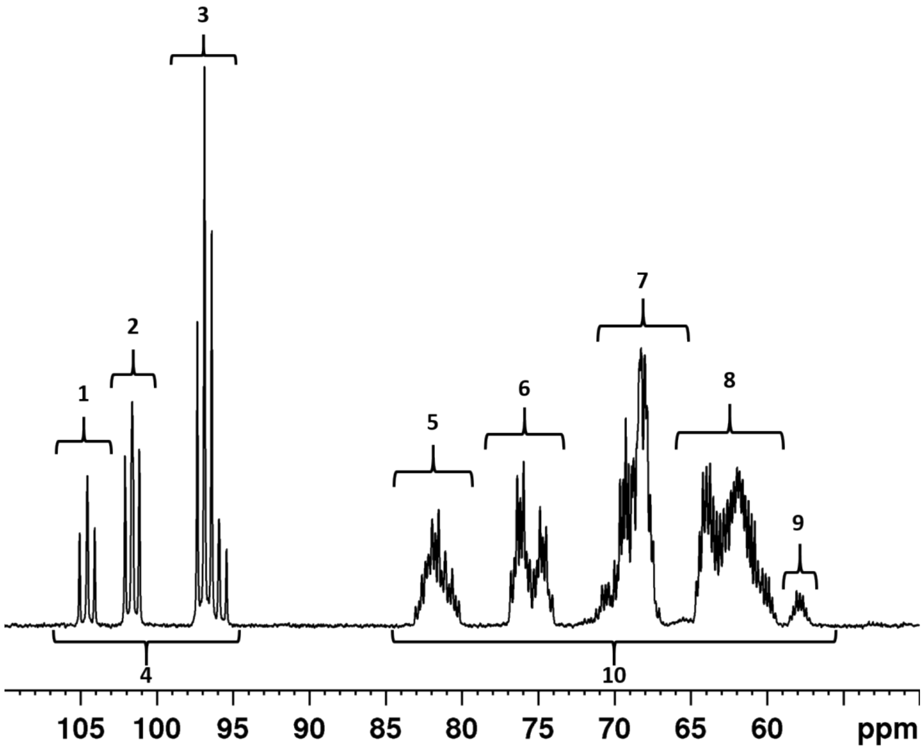

Figure S7 - Hyperpolarized ^{13}C spectrum of a sample containing 4.8 mM of the **3** catalyst with 2.5 mg of D-Fructose-$^{13}C_6$-d_7 in 0.65 ml solvent (DCM:DMF (1.6:1)). Only the region containing the fructose resonances is shown and has been subsequently been labelled with integration regions 1-10 which correspond to the data exhibited in Tables S10 and S11, where peak 11 is all the peaks integrated together.

The hyperpolarized lifetimes have been determined from the data presented in Table S10 and are shown below in Table S11. Peaks 1-4 corresponding to the C-2 resonance in its different isomeric forms all have comparable values around 20 seconds, which is almost double the lifetime of the corresponding glucose molecules. All other resonances are observed to have lifetimes much the same to the D-Fructose-$^{13}C_6$-d_7 molecules analysed.

Table S11 - Hyperpolarized lifetimes (T_1) determined from the values in Table S10 for the different resonances for the sample contained 4.8 mM of the **3** catalyst with 2.5 mg of D-Fructose-$^{13}C_6$-d_7 in 0.65 ml solvent (DCM:DMF (1.6:1)).

Peak #	T_1 (s)	error (s)
1	20.2	0.8
2	20.6	0.8
3	20.4	0.8
4	**20.3**	**0.7**
5	12.1	0.4
6	11	0.3
7	11.3	0.3
8	9.1	0.3
9	9.5	0.4
10	**10.4**	**0.3**
11	**11.7**	**0.3**

Figure S8 shows the hyperpolarized lifetime decay curve for all forms of the C-2 resonance compared with

all other resonances grouped together. The resulting lifetimes for these two curves are 20.3 ± 0.7 s and 10.4 ± 0.3 s for the C-2 and all other resonances respectively.

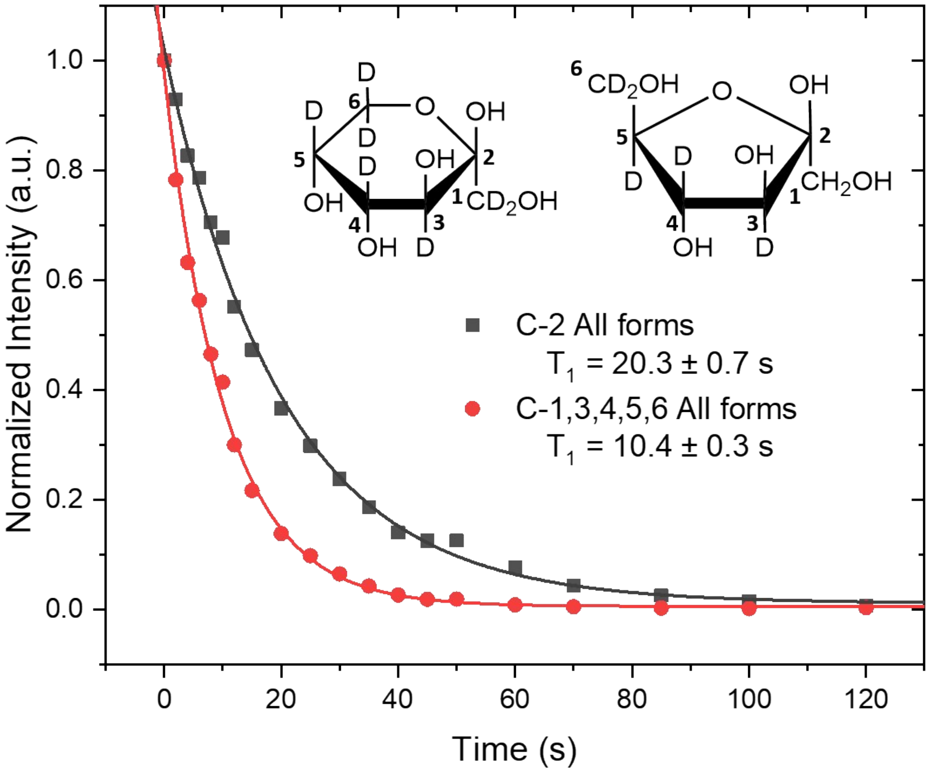

Figure S8 - Hyperpolarized ^{13}C decay curves for C-2 resonance (peak 4 in Table S10) compared to all other resonances combined (peak 10 in Table S10) for the 4.8 mM of the **3** catalyst with 2.5 mg of D-Fructose-$^{13}C_6$-d_7 in 0.65 ml solvent (DCM:DMF (1.6:1)) sample. The structures of the fructopyranose and fructofuranose forms have been included to show the numbering of the carbon nuclei of the fructose molecules.

Fructose Lifetime Measurements without ^{2}H labelling

In the previous section we showed that most of the resonances of the fructose molecule exhibited similar T_1 values to that of the corresponding isotopically labelled glucose molecules, with the exception of the C-2 resonance. The C-2 resonance of fructose is a quaternary carbon and as such has a larger T_1 value than the other resonances. Since there is no directly bonded ^{1}H nucleus on this carbon this resonance should also exhibit a longer T_1 value when the molecule is non-deuterated. In this section we will discuss the hyperpolarized lifetimes determined for the non-deuterated D-Fructose-$^{13}C_6$ sample. A hyperpolarized spectrum of 2.5 mg D-Fructose-$^{13}C_6$ with 4.8 mM of catalyst ***d*** $\boldsymbol{d_{22}}$**-5** in DCM-d_2: DMF (1.6:1) is shown in Figure S9. As with the data presented in the previous section the Fructose measured has 24 different ^{13}C resonances from the different possible isomers. Due to the homonuclear ^{13}C coupling not all of these peaks are resolved and thus in the analysis below we have integrated as many distinguishable peaks as possible. The lifetime measurements here are using the single-shot method which in this instance does not use decoupling, therefore the individual spectra become more strongly coupled. There are two distinct regions that are analysed here, since the C-2 resonance is estimated to have the longest T_1 and has a distinct chemical shift from the other five ^{13}C resonances, the C-2 resonances resulting from all isomers of the fructose have been analysed together (peak 1) and the other resonances have also been grouped together (peak 2). Since there is a significant difference between the observed T_1 for each of these grouped resonances two different measurements had to be acquired with different spacing of the FIDs in order to capture the entire decay of both resonances, as shown in Table S12.

Table S12 - Intensities of the ^{13}C resonances detailed in Figure S9 for all C-2 resonances of fructose and all other ^{13}C grouped together (peak 2) as a function of time after the hyperpolarization step (where $t = 0$ depicts the moment after transfer to the spectrometer). The sample contained 4.8 mM of the $\boldsymbol{d_{22}}$**-5** catalyst with 2.5 mg of D-Fructose-$^{13}C_6$ in 0.65 ml solvent (DCM:DMF (1.6:1)). For each peak they were relaxing at different rates so separate single-shot sequences were employed with different FID spacing in each case, shown below.

	Peak 1 Normalized Intensity (a.u.)					Peak 2 Normalized Intensity (a.u.)			
Time (s)	1	2	3	Accum.	Time (s)	1	2	3	Accum.
0.00	1.000	1.000	1.000	1.000	0.00	1.000	1.000	1.000	1.000
4.45	0.684	0.679	0.703	0.688	0.70	0.731	0.740	0.726	0.733
8.90	0.506	0.524	0.511	0.514	1.40	0.529	0.528	0.507	0.522
13.35	0.374	0.350	0.342	0.355	2.10	0.372	0.381	0.374	0.376
17.80	0.247	0.243	0.238	0.243	2.80	0.259	0.276	0.257	0.264
22.25	0.158	0.168	0.181	0.169	3.50	0.184	0.190	0.171	0.182
26.70	0.129	0.120	0.144	0.131	4.20	0.128	0.128	0.132	0.129
31.15	0.082	0.077	0.090	0.083	4.90	0.084	0.085	0.090	0.086
35.60	0.060	0.057	0.058	0.058	5.60	0.052	0.066	0.056	0.058
40.05	0.034	0.028	0.037	0.033	6.30	0.041	0.044	0.039	0.041
44.50	0.035	0.036	0.016	0.029	7.00	0.026	0.032	0.026	0.028
48.95	0.038	0.015	0.028	0.027	7.70	0.018	0.023	0.018	0.020
53.40	-0.004	0.015	0.014	0.008	8.40	0.017	0.015	0.010	0.014
57.85	0.012	0.017	0.012	0.013	9.10	0.020	0.020	0.011	0.017
62.30	0.015	0.013	0.017	0.015	9.80	0.017	0.015	0.014	0.015

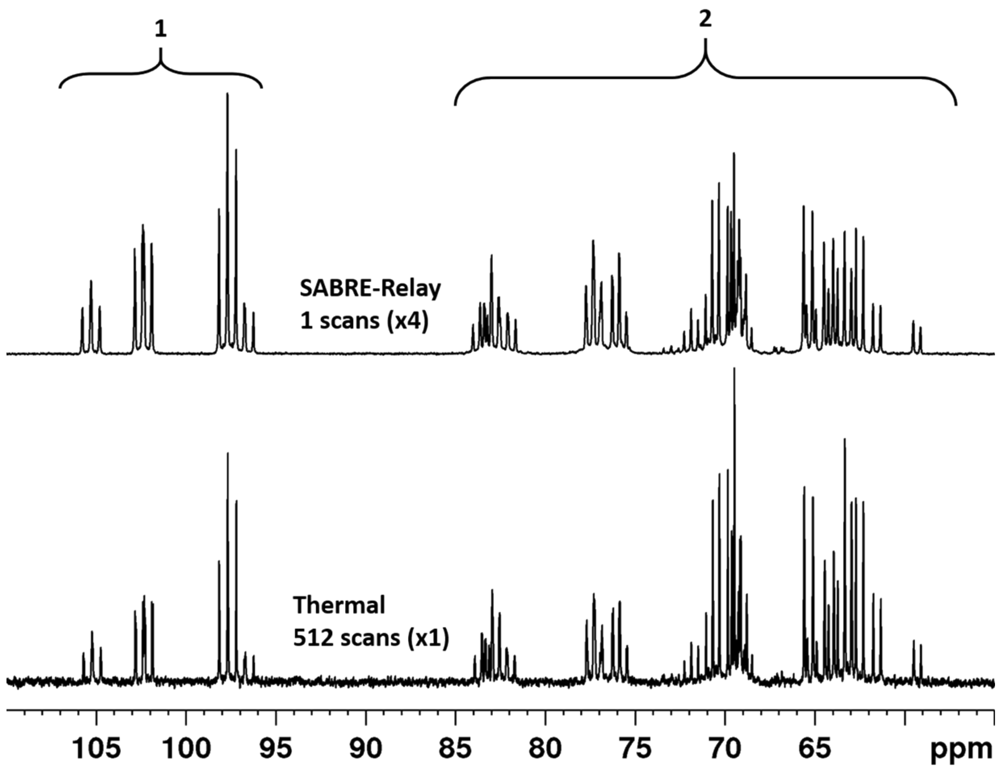

Figure S9 - Hyperpolarized $^{13}C\{^{1}H\}$ spectrum (top, x4) and thermal reference spectrum (bottom, 512 scans) of a sample containing 4.8 mM of the ***d*₂₂-5** catalyst with 2.5 mg of D-Fructose-$^{13}C_6$ in 0.65 ml solvent (DCM:DMF (1.6:1)). Only the region containing the fructose resonances has been shown and has been subsequently been labelled with integration regions 1 and 2 which correspond to the data displayed in Tables S12 and S13.

The hyperpolarized lifetimes have been determined from the data presented in Table S12 and are shown below in Table S13. Peaks 1 and 2 correspond to the C-2 resonance in its different isomeric forms and C-1,3,4,5,6, respectively. The C-2 resonance here is shown to have a hyperpolarized lifetime of 12.8 ± 0.2 seconds which is comparable to the deuterated form of glucose, however here there was no need for expensive deuterium labelling. All other resonances are observed to have lifetimes comparable to the corresponding D-Glucose-$^{13}C_6$ molecules analysed, giving an average value of 2.11 ± 0.03 seconds. In each case three repeat single-shot experiments were carried out optimized for each resonance. All three repeats are shown as well as the accumulated data of all three individual runs.

Table S13 - Hyperpolarised lifetimes (T_1) determined from the values in Table S12 for the different resonances for the sample contained 4.8 mM of the **d_{22}-5** catalyst with 2.5 mg of $^{13}C_6$-Fructose in 0.65 ml solvent (DCM:DMF (1.6:1)).

	T_1 (s)	error
1	12.9	0.4
2	12.8	0.3
3	12.8	0.3
Accum.	12.8	0.2
1	2.10	0.04
2	2.15	0.04
3	2.09	0.03
Accum.	2.11	0.03

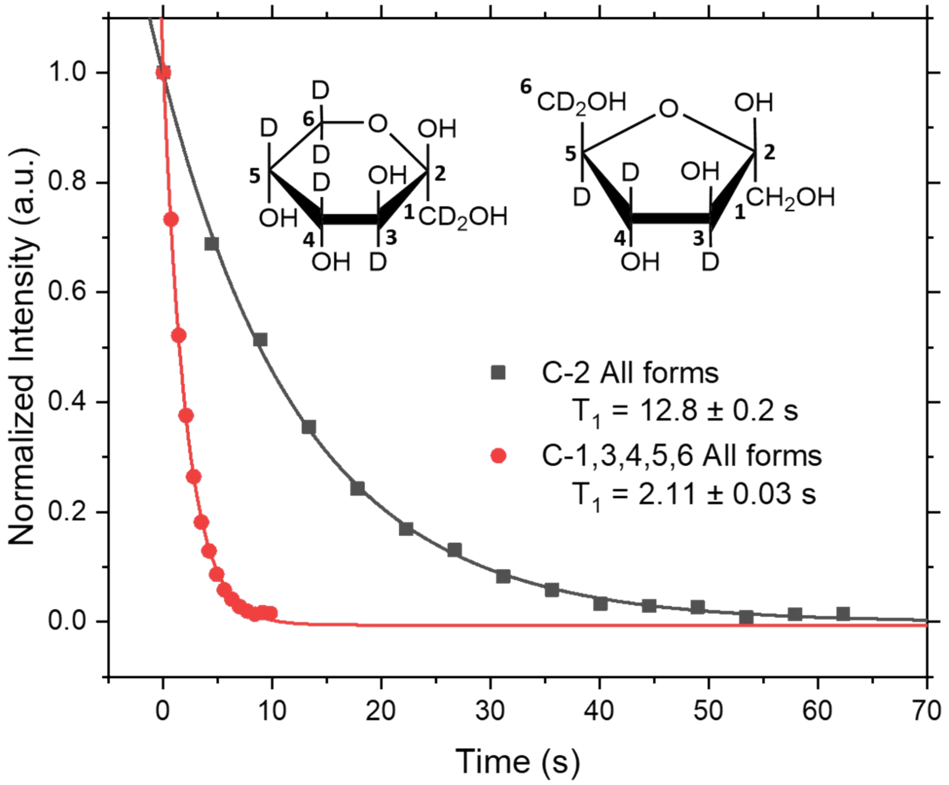

Figure S10 - Hyperpolarized ^{13}C decay curves for C-2 resonance (peak 1 in Table S12 and S13) compared to all other resonances combined (peak 2 in Table S12 and S13) for the 4.8 mM of the $\boldsymbol{d_{22}}$**-5** catalyst with 2.5 mg of D-Fructose-$^{13}C_6$ in 0.65 ml solvent (DCM:DMF (1.6:1)) sample. The structures of the fructopyranose and fructofuranose forms have been included to show the numbering of the carbon nuclei of the fructose molecules.

SABRE-Relay Shake Time Dependence

Figure 6e of the main text shows the SABRE-Relay polarization achieved as a function of time shaken in the PTF field. In order to achieve these data, the sample was refilled with 4 bar (absolute) of *para*-hydrogen after evacuation of the headspace and subsequently shaken for a given time (τ). A sample 2.5 mg D-Glucose-$^{13}C_6$-d_7 with 4.8 mM of ***d*₂₂-5** in DCM: DMF (1.6: 1) was used. The data presented in Figure 6e show that there is a clear maximum with time showing an initial increase with time, followed by a subsequent decrease. The initial increase is attributed to the build-up of polarization which is believed to be linked to the T_1 of the molecule, with the decrease being attributed to the overall consumption of *para*-hydrogen and T_1. In the case where *para*-hydrogen is not limited it would be expected that the polarization would increase and eventually plateau with increasing time, as an equilibrium would be reached whereby the polarization gained would be offset by the relaxation. It was possible to quantify these two processes shown in Figure 6e by fitting the data to an empirical bi-exponential function, with an exponential term for each of the processes. Equation S5 shows the dependence of the enhancement factor (ε) as a function of shake time (τ) with ε_0, A_1, t_1, A_2 and t_2 being the exponential fitting parameters.

$$\varepsilon(\tau) = \varepsilon_0 + A_1 e^{-\frac{\tau}{t_1}} - A_2 e^{-\frac{\tau}{t_2}} \qquad \text{(S5)}$$

The first exponential term describes the build-up of polarization and with the second term representing the decay which has been attributed to the loss of *para*-hydrogen over the experiment since it is being carried out in a tube, with a finite source. Since equation 1 represents a peak, the shake time that yields the largest enhancement (τ_{max}) can be determined by first differentiating equation 1 and setting the differential to zero to find the turning point, upon rearranging yields equation S6.

$$\tau_{max} = \frac{\ln\left(\frac{A_1 t_2}{A_2 t_1}\right)}{\left(\frac{1}{t_1} - \frac{1}{t_2}\right)} \qquad (S6)$$

The parameters determined from the data presented in Figure 6e can be found Table S14 and yield a maximum enhancement when the shake time is 15.3 seconds.

Table S14 – Parameters determined from the fitting of equation S5 to the data displayed in Figure 6e of the main text. These are derived from the semi0empirical equation employed here (Equation S5) to determine the optimal shake time.

Parameter	Value
ε_0	52.01
A_1	393.7
t_1 (s)	37.6
A_2	429.6
t_2 (s)	7.06

Isotopic Labelled Glucose Isomer Analysis

It was possible to determine the proportion of the different isomers present in the overall solution from the hyperpolarized signals. The resonances for the C-1 resonance for both the α and β forms are distinguishable when detected at 9.4 T which allows the proportion of them to be determined as per equation S7.

$$\alpha(\%) = \frac{I_\alpha}{I_\alpha + I_\beta} \quad and \quad \beta(\%) = \frac{I_\beta}{I_\alpha + I_\beta} \qquad (S7)$$

Where the NMR intensity of the alpha and beta isomers are represented by I_α and I_β respectively. The data acquired for 10 seconds of shaking in 60 G PTF using 4.8 mM of the ***d*₂₂-5** catalyst with 2.5 mg of D-Glucose-$^{13}C_6$-d_7 in 0.65 ml solvent (DCM:DMF (1.6:1)) is displayed in table S15. Table S15 also shows the calculated proportions as a percentage of each isomer over the 5 repeat measurements along with the average values. Table S16 shows the corresponding measurement of the thermal magnetisation of the same sample as discussed in Table S15.

Table S15 - Intensities of the C-1 resonance for the α-glucose and β-glucose isomers determined for several repeat measurements for 10 seconds of shaking with 4 bar (absolute) *para*-hydrogen gas in 60 G PTF using a sample containing 4.8 mM of the **d_{22}-5** catalyst with 2.5 mg of D-Glucose-$^{13}C_6$-d_7 in 0.65 ml solvent (DCM:DMF (1.6:1)).

Exp.	1	2	3	4	5	average	error
I_β	19081237	19984120	19808743	20226267	20007940	19738665	180725
I_α	16909147	17917129	17515095	17472728	18033815	17473028	188371
β (%)	53.0	52.7	53.1	53.7	52.6	53.0	0.2
α (%)	47.0	47.3	46.9	46.3	47.4	47.0	0.2

Table S16 - Intensities of the C-1 resonance for the alpha and beta isomers of glucose determined for a thermal magnetization measurement of the same sample containing 4.8 mM of the **d_{22}-5** catalyst with 2.5 mg of D-Glucose-$^{13}C_6$-d_7 in 0.65 ml solvent (where a ratio of solvents DCM:DMF (1.6:1) was used) acquired with 512 scans.

Exp.	1
I_β	36746553
I_α	30583947
β (%)	54.57638
α (%)	45.42362

Table S15 shows that on average the amount of β-glucose and α-glucose is 53.0 ± 0.2 % and 47. 0 ± 0.2 % respectively, as determined from the hyperpolarized measurements. The distribution obtained from the thermal reference gave 54.6 % and 45.4 % for the β-glucose and α-glucose respectively. Therefore, the distribution from the hyperpolarized is comparable to the thermal case and therefore can be assumed that the hyperpolarized result can yield the isomer distribution in a matter of seconds.

Natural Abundance Glucose Isomer Analysis

In the previous section it was shown that the different isomeric forms of ^{13}C labelled glucose in solution could be determined from a single SABRE-Relay hyperpolarization step. A natural abundance D-glucose sample has also been analysed under the same conditions, namely with 40 mM of D-glucose, 4.8 mM of the ***d*₂₂-5** catalyst, 26 mM benzyl-d_7-amine and DCM-d_2: DMF (1.6:1). 16 repeat polarization experiments were carried out, where the corresponding integrals and subsequent calculations of the percentages of each isomer are displayed in Table S17. Due to the significant reduction in signal to noise that is observed when transitioning from the isotopically labelled to natural abundance forms of glucose the FIDs of the 16 scans were accumulated and also processed and displayed in Table S17. Additionally, since SABRE-Relay has been shown to be highly reproducible between subsequent hyperpolarization steps, this accumulation is synonymous with signal averaging, and thus should allow the retention of quantitative nature of conventional NMR coupled with the high signal of a hyperpolarized technique.

Table S17 - Intensities of the C-1 resonance for the α-D-glucose and β-D-glucose isomers of glucose determined for several repeat measurements for 10 seconds of shaking in 60 G PTF using a sample containing 4.8 mM of the **d_{22}-5** catalyst with 40 mM of natural abundance D-glucose in 0.65 ml solvent (where a ratio of solvents DCM:DMF (1.6:1) was used). The FIDs of the 16 scans were accumulated and the integrals were also compared to give a greater signal to noise.

Exp. #	I_α	I_β	α (%)	β (%)
1	38939	39518	49.63	50.37
2	41149	52011	44.17	55.83
3	47418	62822	43.01	56.99
4	37317	60123	38.30	61.70
5	44945	61830	42.09	57.91
6	42781	55621	43.48	56.52
7	44236	61196	41.96	58.04
8	36201	67098	35.05	64.95
9	38249	72746	34.46	65.54
10	44292	56390	43.99	56.01
11	57006	56478	50.23	49.77
12	57877	59516	49.30	50.70
13	31628	55222	36.42	63.58
14	54902	67867	44.72	55.28
15	45857	50307	47.69	52.31
16	45101	54159	45.44	54.56
average	44244	58307	**43.12**	**56.88**
error	1853	1959	1.25	1.25
error %	4.2	3.4	2.9	2.2
accumulated	741513	916784	**44.72**	**55.28**

The variation between hyperpolarization scans was fairly significant which was attributed to the low signal to noise on a single hyperpolarized scan using a natural abundance sample. However, the signal to noise of the accumulated scans was much better and gave a more reliable result, which gave the two isomeric forms to be 44.72 % and 55.28 % for the α-D-glucose and β-D-glucose respectively. The values obtained here are comparable to the results observed for the isotopically

labelled version shown previously of 47. 0 ± 0.2 % and 53.0 ± 0.2 %. Therefore, it seems feasible to be able to determine the isomeric distribution of natural abundance glucose in low concentration solutions in a matter of minutes. The corresponding thermal reference scan at this concentration would take on the order of days to be able to determine these distributions accurately. An example single scan spectrum and the accumulated FID derived spectrum are shown in Figure S11 to highlight the signal to noise benefits of signal averaging with SABRE-Relay hyperpolarization.

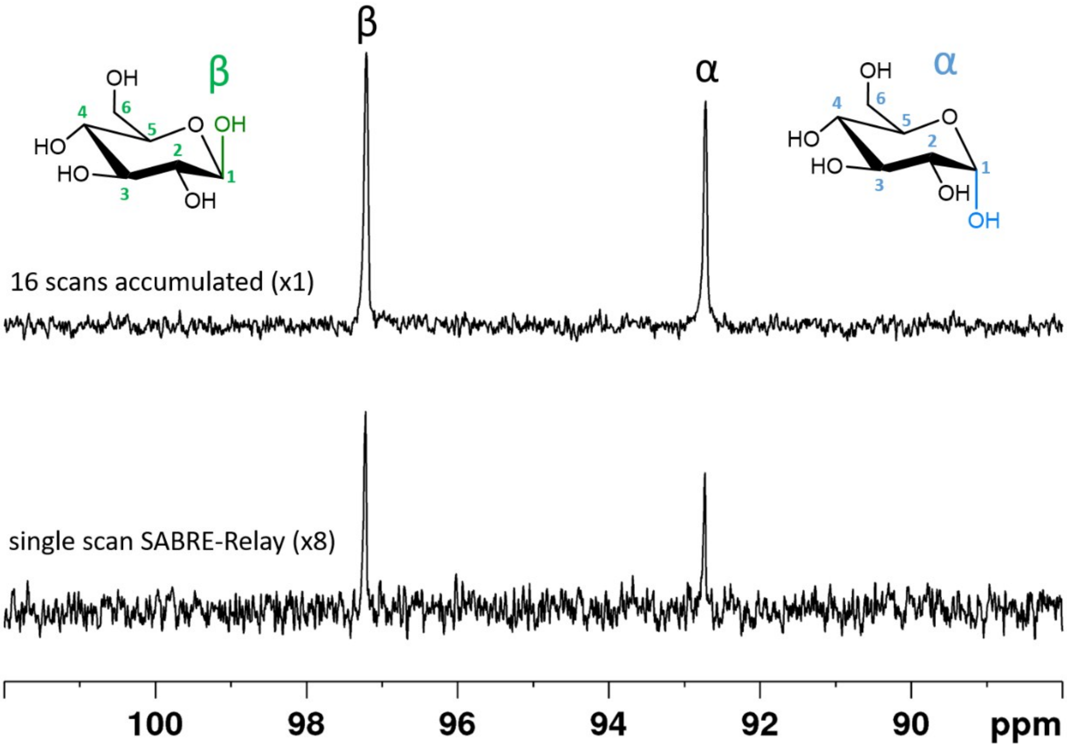

Figure S11 - Single hyperpolarization step (bottom spectrum) and a spectrum derived from 16 accumulated hyperpolarized FIDs (top spectrum) for the C-1 resonance for the α-D-glucose and β-D-glucose isomers of glucose for 10 seconds of shaking in 60 G PTF using a sample containing 4.8 mM of the ***d*₂₂-5** catalyst with 40 mM of natural abundance D-glucose in 0.65 ml solvent (where a ratio of solvents DCM:DMF (1.6:1) was used). Sample headspace was evacuated and refilled with fresh *para*-hydrogen between each hyperpolarization step.

Isotopic Labelled Fructose Isomer Analysis

Similar to the case of the glucose, the fructose also contains different tautomers in solution. Much like the glucose it was also possible to determine the proportion of these different isomers present in the overall solution from a single hyperpolarized scan. Unlike the glucose, the fructose has four distinguishable isomers as it can form both a five-membered ring (fructofuranose) and a six-membered ring (fructopyranose), which can both in turn form the α and β isomers. Therefore, in order to determine the percentage of each form the following equations can be used. It is important to note that the keto form can also be present in solution, however, typically this is in negligible amounts.[2-3]

$$\alpha fur(\%) = \frac{I_{\alpha f}}{I_{\alpha f} + I_{\beta f} + I_{\alpha p} + I_{\beta p}} \quad and \quad \beta fur(\%) = \frac{I_{\beta f}}{I_{\alpha f} + I_{\beta f} + I_{\alpha p} + I_{\beta p}} \qquad (S8)$$

$$\alpha pyr(\%) = \frac{I_{\alpha p}}{I_{\alpha f} + I_{\beta f} + I_{\alpha p} + I_{\beta p}} \quad and \quad \beta pyr(\%) = \frac{I_{\beta p}}{I_{\alpha f} + I_{\beta f} + I_{\alpha p} + I_{\beta p}}$$

Where the NMR intensity of the α-fructofuranose, β-fructofuranose, α-fructopyranose, β-fructopyranose are represented by $I_{\alpha f}$, $I_{\beta f}$, $I_{\alpha p}$ and $I_{\beta p}$ respectively. The data acquired for 10 seconds of shaking in a 60 G PTF using 4.8 mM of the ***d*$_{22}$-5** catalyst with 2.5 mg of D-Fructose-$^{13}C_6$-d_7 in 0.65 ml solvent (where a ratio of solvents DCM:DMF (1.6:1) was used) and 4 bar (absolute) *para*-hydrogen is displayed in table S18. The percentage of each isomer over 5 repeat measurements are shown. Table S19 shows the corresponding measurement of the thermal magnetisation of the same sample as discussed in Table S18.

Table S18 - Intensities of the C-2 resonance for the α-fructofuranose, β-fructofuranose, α-fructopyranose, β-fructopyranose isomers of fructose determined for several repeat measurements for 10 seconds of shaking in 60 G PTF using a sample containing 4.8 mM of the ***d*$_{22}$-5** catalyst with 2.5 mg of D-Fructose-$^{13}C_6$-d_7 in 0.65 ml solvent (where a ratio of solvents DCM:DMF (1.6:1) was used).

Exp.	1	2	3	4	5	Average	error
I-αf	4446243	4559180	4568403	4383112	4630557	4517499	44860
I-βf	8669921	8899602	8926362	8580359	9051918	8825632	86956
I-αf	3428102	3466388	3493481	3345442	3527443	3452171	31261
I-βp	12879278	13231897	13324320	12680477	13406465	13104488	138970
α-f (%)	15.11	15.12	15.07	15.12	15.12	**15.11**	0.01
β-f (%)	29.47	29.51	29.45	29.60	29.57	**29.52**	0.03
α-p (%)	11.65	11.49	11.52	11.54	11.52	**11.55**	0.03
β-p (%)	43.77	43.88	43.96	43.74	43.79	**43.83**	0.04

In the hyperpolarized spectrum for the fructose molecule the α and β-fructopyranose peaks partially overlap, with the outer part of the triplet in each case overlapping. The triplet is observed due to the coupling to two adjacent ^{13}C labelled nuclei in the structure. In order to determine the individual integrals for the two peaks, the distinguishable two thirds of each triplet was integrated and subsequently scaled up by a factor 4/3 as the triplets all have a standard 1-2-1 integration pattern. This method was checked by doing this method and checking to see if the total integral of the peak was equal to the summation of the individual peaks. The peaks are shown in Figure S12, highlighting which peak corresponds to which isomeric form.

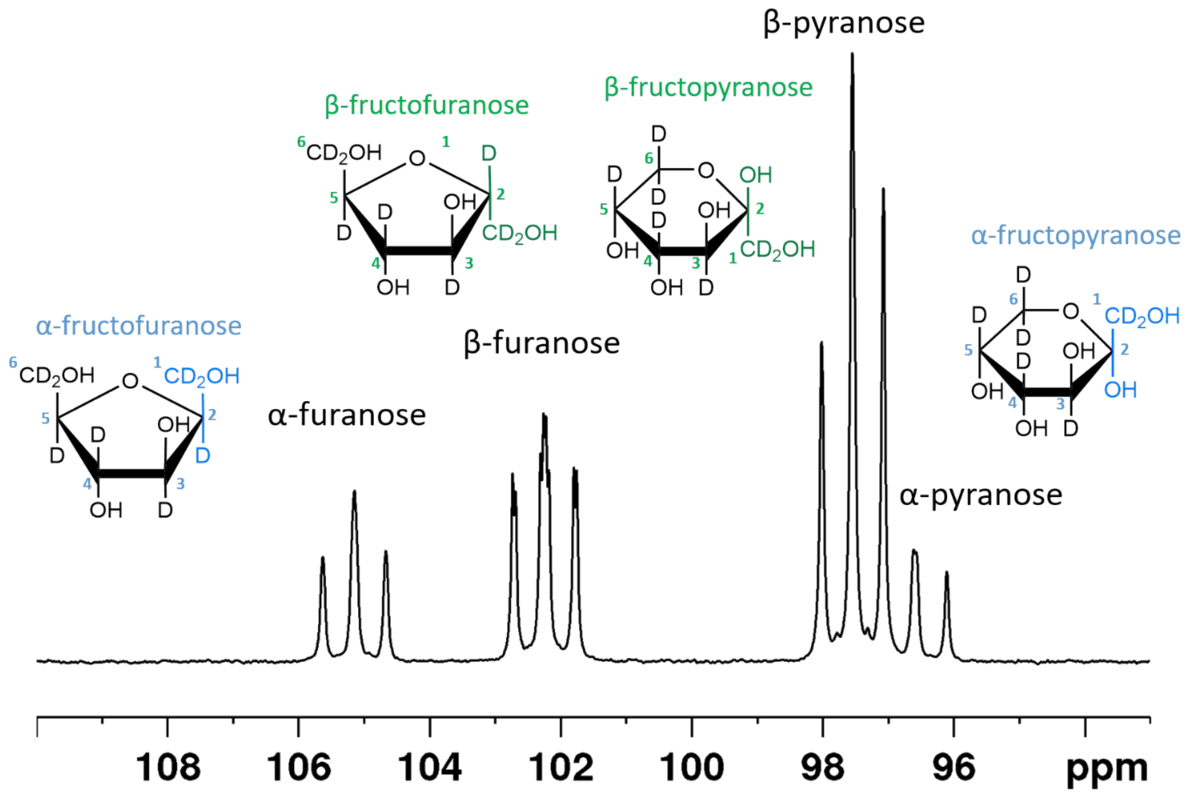

Figure S12 – Hyperpolarized spectrum for a sample containing 4.8 mM ***d*₂₂-5** catalyst with 2.5 mg of D-Fructose-$^{13}C_6$-d_7 with 26 mM benzyl-d_7-amine in DCM-d_2: DMF (1,6:1). The region of interest shown is the C-2 position of the fructofuranose and fructopyranose. The insets show colour coded structures with α forms being shown in blue and β forms in green, with both the five-membered furanose and six-membered pyranose motifs.

Table S19 - Intensities of the C-2 resonance for the α-fructofuranose, β-fructofuranose, α-fructopyranose and β-fructopyranose isomers of fructose determined from a thermal magnetization measurement of the same sample containing 4.8 mM of the **d_{22}-5** catalyst with 2.5 mg of D-Fructose-$^{13}C_6$-d_7 in 0.65 ml solvent (DCM:DMF (1.6:1)) acquired with 512 scans with a total experiment time of around 18 hours.

Exp.	*1*
I-αf	12102120
I-βf	25812879
I-αp	9411841
I-βp	40147204
αf (%)	**13.84**
βf (%)	**29.51**
αp (%)	**10.76**
βp (%)	**45.90**

Table S19 shows that on average the amounts of α-fructofuranose, β-fructofuranose, α-fructopyranose and β-fructopyranose were 15.11 ± 0.01 %, 29.52 ± 0.03 %, 11.55 ± 0.03 % and 43.83 ± 0.04 % respectively, as determined from the hyperpolarized measurements. The corresponding distributions observed from the thermal reference gave 13.84 %, 29.51 %, 10.76 % and 45.90 % for the α-fructofuranose, β-fructofuranose, α-fructopyranose and β-fructopyranose respectively. The distribution determined from the hyperpolarized and thermal reference spectra are comparable

with the variations being -8.5 %, -0.1 %, +4.7 % and -6.4 % for the α-fructofuranose, β-fructofuranose, α-fructopyranose and β-fructopyranose respectively. This difference could be due to signal to noise as the peaks with the greatest deviance are those with the smaller integrals, where the hyperpolarised measurements provide significantly larger signal to noise measurements in 10 seconds as opposed to the 18 hour thermal acquisition. The thermal reference spectrum used to determine the values in Table S19 is shown in Figure S13, highlighting the region of interest for the C-2 resonance for the four isomeric forms of fructose.

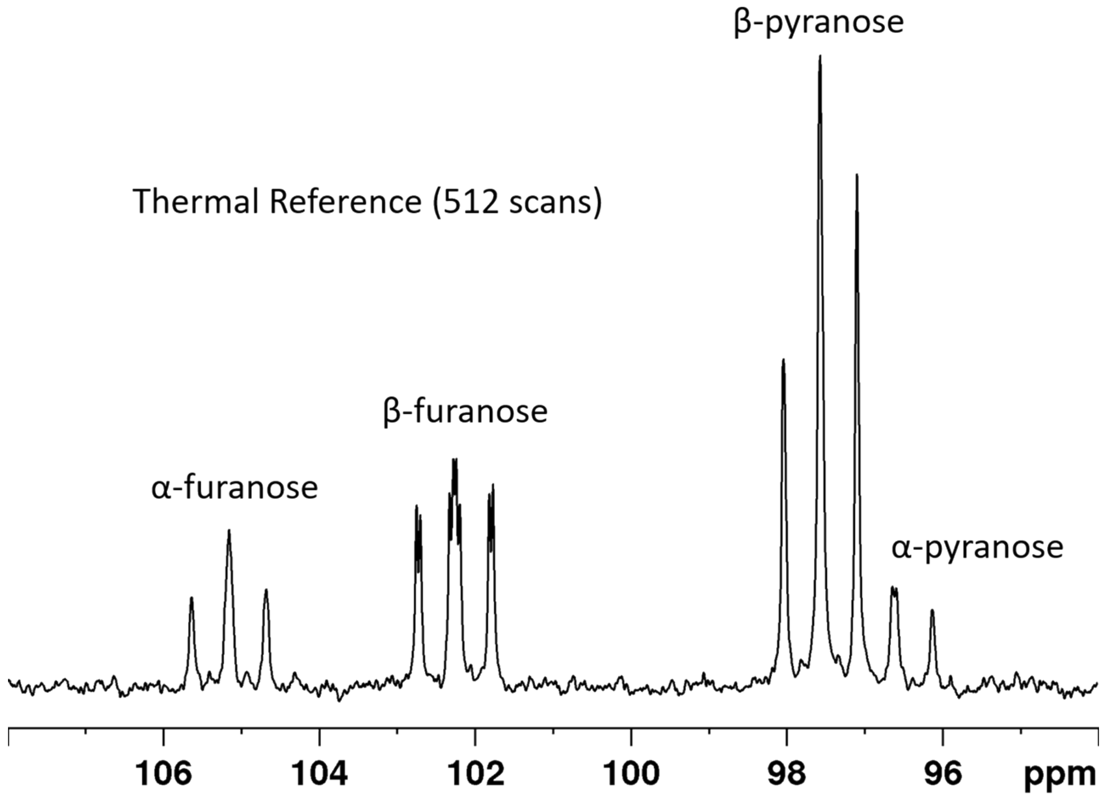

Figure S13 – Thermal reference spectrum for a sample containing 4.8 mM ***d*$_{22}$-5** catalyst with 2.5 mg of D-Fructose-$^{13}C_6$-d_7 with 26 mM benzyl-d_7-amine in DCM-d_2: DMF (1.6:1). The region of interest shown is the C-2 resonance of the fructofuranose and fructopyranose.

Natural Abundance Fructose Isomer Analysis

In the previous section the isotopically labelled ^{13}C and ^{2}H D-Fructose was shown to give the percentage of tautomers in solution in a single hyperpolarization experiment, which was comparable to a thermal reference scan. In this section we assess the viability of being able to determine the four dominant tautomers of fructose using a natural abundance sample. A percentage of each of the four isomers was determined for each hyperpolarization step which are displayed in Table S20. Also included in Table S20 are the distributions arising from the accumulation of the FIDs of the 16 repeat hyperpolarization runs. Due to the low signal to noise produced by a single hyperpolarization step the individual distributions are quite scattered. However, the signal to noise obtained when signal averaging with SABRE-Relay is sufficient to obtain a reliable isomeric distribution. The distribution was seen to give 15.48 %, 32.89 %, 11.21 % and 40.42 % for the α-fructofuranose, β-fructofuranose, α-fructopyranose and β-fructopyranose respectively. These numbers are comparable to the ones observed for the isotopically labelled sample. This suggests that an estimation of natural abundance ^{13}C fructose can be analysed in a matter of minutes compared to the order of several days to achieve the corresponding thermal reference at this concentration.

Table S20 - Intensities of the C-2 resonance for the α-fructofuranose, β-fructofuranose, α-fructopyranose, β-fructopyranose isomers of fructose determined for 16 repeat measurements for 10 seconds of shaking in 60 G PTF using a sample containing 4.8 mM of the ***d***$\boldsymbol{d_{22}}$**-5** catalyst with 40 mM natural abundance D-Fructose in 0.65 ml solvent (where a ratio of solvents DCM:DMF (1.6:1) was used). Due to low signal to noise the FIDs of the 16 scans were accumulated, essentially signal averaging with SABRE-Relay which gave a more accurate analysis.

Exp. #	I-αf	I-βf	I-αp	I-βp	αf (%)	βf (%)	αf (%)	βp (%)
1	47938.78	97376.24	17108.52	121494.38	16.88	34.30	6.03	42.79
2	43652.7	101534.91	26589.01	120564.51	14.93	34.73	9.10	41.24
3	49203.66	111118.45	21757.56	121514.87	16.21	36.60	7.17	40.03
4	54192.28	94800.31	22467.17	132749.66	17.81	31.16	7.39	43.64
5	46636.49	99801.46	38519.03	128403.25	14.88	31.85	12.29	40.98
6	52046.87	110727.04	35765.51	118465.61	16.42	34.93	11.28	37.37
7	52200.19	111623.69	21962.83	143421.98	15.86	33.91	6.67	43.57
8	48865.85	105857.38	44014.79	140118.64	14.42	31.24	12.99	41.35
9	60910.43	112974.01	39461.5	136749.97	17.40	32.27	11.27	39.06
10	52535.73	121604.74	38333.36	125061.06	15.56	36.03	11.36	37.05
11	50595.94	117838.28	39186.23	156275.09	13.90	32.38	10.77	42.95
12	50310.94	98479.11	44901.74	139132.88	15.12	29.59	13.49	41.80
13	46838.39	104821.43	39605	145113.03	13.92	31.16	11.77	43.14
14	49216.61	116123.64	32870.89	130126.36	14.99	35.37	10.01	39.63
15	49012.29	114152.16	38679.26	135429.06	14.53	33.85	11.47	40.15
16	53533.2	94128.18	34144.16	127349.58	17.32	30.45	11.04	41.19
Ave.	50481	107060	33460	132623	**15.64**	**33.11**	**10.26**	**41.00**
error	980.4	2161	2176	2628	0.31	0.53	0.58	0.51
%error	1.9	2.0	6.5	1.98	2.01	1.60	5.63	1.24
Acc. data	818388	1738614	592410	2136977	**15.48**	**32.89**	**11.21**	**40.42**

Figure S14 shows the hyperpolarized spectrum of 4.8 mM of the ***d***$\boldsymbol{d_{22}}$**-5** catalyst with 40 mM natural abundance D-Fructose in DCM-d_2: DMF (1.6:1). The bottom spectrum shows a single scan SABRE-Relay hyperpolarization step, with the top spectrum showing the 16 accumulated hyperpolarization steps. Figure S14 also shows the structures of the four different isomeric forms of the D-Fructose.

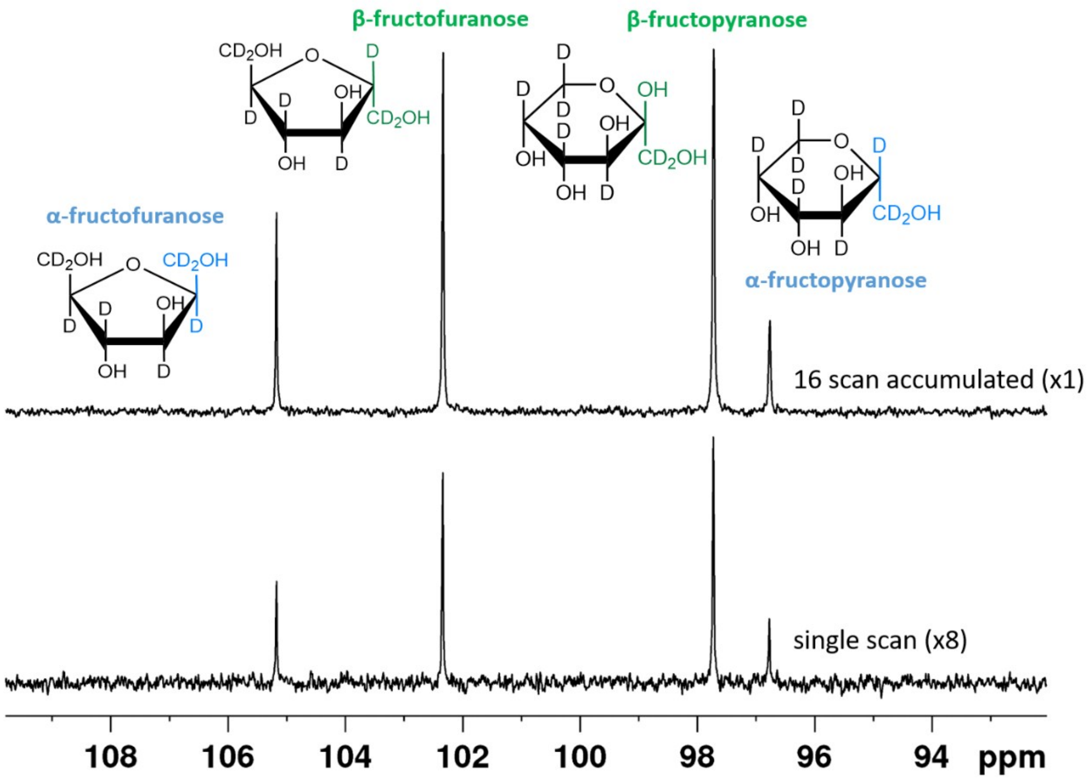

Figure S14 - Hyperpolarized spectrum of 4.8 mM of the $\boldsymbol{d_{22}}$**-5** catalyst with 40 mM natural abundance D-Fructose in DCM-d_2: DMF (1.6:1) in a single hyperpolarization step (bottom) and after 16 accumulated hyperpolarization steps (top). Structures of the four isomeric forms of D-Fructose have been included.

References

1. Pfeffer, P. E.; Valentine, K. M.; Parrish, F. W., Deuterium-Induced Differential Isotope Shift C-13 Nmr .1. Resonance Re-Assignments of Monosaccharides and Disaccharides. *J. Am. Chem. Soc.* **1979,** *101* (5), 1265-1274.

2. Flood, A. E.; Johns, M. R.; White, E. T., Mutarotation of D-fructose in aqueous-ethanolic solutions and its influence on crystallisation. *Carbohydr. Res.* **1996,** *288*, 45-56.

3. Barclay, T.; Ginic-Markovic, M.; Johnston, M. R.; Cooper, P.; Petrovsky, N., Observation of the keto tautomer of D-fructose in D2O using H-1 NMR spectroscopy. *Carbohydr. Res.* **2012,** *347* (1), 136-141.
